# Optimal Hypoxia Regulates Human iPSC-Derived Liver Bud Differentiation through Intercellular TGFB Signaling

**DOI:** 10.1016/j.stemcr.2018.06.015

**Published:** 2018-07-19

**Authors:** Hiroaki Ayabe, Takahisa Anada, Takuo Kamoya, Tomoya Sato, Masaki Kimura, Emi Yoshizawa, Shunyuu Kikuchi, Yasuharu Ueno, Keisuke Sekine, J. Gray Camp, Barbara Treutlein, Autumn Ferguson, Osamu Suzuki, Takanori Takebe, Hideki Taniguchi

**Affiliations:** 1 Department of Regenerative Medicine, Yokohama City University Graduate School of Medicine, Kanazawa-ku 3-9, Yokohama, Kanagawa 236-0004, Japan; 2 Division of Craniofacial Function Engineering, Tohoku University Graduate School of Dentistry, 4-1 Seiryo-machi, Aoba-ku, Sendai 980-8575, Japan; 3 Max Planck Institute for Evolutionary Anthropology, Leipzig 04103, Germany; 4 Division of Gastroenterology, Hepatology & Nutrition, Developmental Biology, Center for Stem Cell and Organoid Medicine (CuSTOM), Cincinnati Children's Hospital Medical Center, 3333 Burnet Avenue, Cincinnati, OH 45229-3039, USA; 5 Department of Pediatrics, University of Cincinnati College of Medicine, 3333 Burnet Avenue, Cincinnati, OH 45229-3039, USA; 6 Institute of Research, Tokyo Medical and Dental University, 1-5-45 Yushima, Bunkyo-ku, Tokyo 113-8510, Japan

**Keywords:** liver bud, organogenesis, organoid, oxygen, iPSC, differentiation, hypoxia

## Abstract

Timely controlled oxygen (O_2_) delivery is crucial for the developing liver. However, the influence of O_2_ on intercellular communication during hepatogenesis is unclear. Using a human induced pluripotent stem cell-derived liver bud (hiPSC-LB) model, we found hypoxia induced with an O_2_-permeable plate promoted hepatic differentiation accompanied by *TGFB1* and *TGFB3* suppression. Conversely, extensive hypoxia generated with an O_2_-non-permeable plate elevated *TGFBs* and cholangiocyte marker expression. Single-cell RNA sequencing revealed that *TGFB1* and *TGFB3* are primarily expressed in the human liver mesenchyme and endothelium similar to in the hiPSC-LBs. Stromal cell-specific RNA interferences indicated the importance of TGFB signaling for hepatocytic differentiation in hiPSC-LB. Consistently, during mouse liver development, the *Hif1a*-mediated developmental hypoxic response is positively correlated with *TGFB1* expression. These data provide insights into the mechanism that hypoxia-stimulated signals in mesenchyme and endothelium, likely through TGFB1, promote hepatoblast differentiation prior to fetal circulation establishment.

## Highlights

• Mesenchyme (MC) and endothelium (EC) in human liver buds primarily express TGFBs
• Hypoxia modulates TGFBR ligand expressions
• TGFB suppression in EC and MC promotes hepatoblast differentiation
• Optimal hypoxia-stimulated TGFB signal promotes hepatoblast differentiation

## Teaser

To delineate the influence of O_2_ on liver development, human induced pluripotent stem cell-derived liver buds were used as a fetal liver model. This model provides insight into hypoxic modulation of mesenchyme-derived TGFB signals that promote hepatoblast differentiation in developing liver buds prior to blood circulation.

## Introduction

Organoid technology using human pluripotent stem cells or isolated organ progenitors is rapidly evolving to model the elaborate spatiotemporal processes of development and regeneration. Progenitors are directed to self-organize into an organoid comprising multiple cell types, including epithelial and mesenchymal cell lineages (Lancaster and Knoblich, 2014). Because human organoids recapitulate certain aspects of native organ architecture that are highly inaccessible, organoid technology has recently emerged as an essential tool for both human biology and pathology research (Kretzschmar and Clevers, 2016). For example, a human induced pluripotent stem cell-derived liver bud (hiPSC-LB) can be generated by co-culture with hiPSC-derived liver progenitor cells, human umbilical vein endothelial cells (HUVECs), and human mesenchymal stem cells (MSCs) (Takebe et al., 2013 and 2017). The complex self-organized hiPSC-LB is an organ-like 3D tissue with cell-cell interactions that recapitulate early LB structures and transcriptomic signatures (Camp et al., 2017). However, *in vitro* organoids generally remain developmentally immature without transplantation into animals (Dye et al., 2016, Xinaris et al., 2012, Watson et al., 2014), highlighting the need for filling the maturation gaps between *in vitro* and *in vivo* systems.

Developmental hypoxia is considered a critical phenomenon, especially in early phases of organogenesis prior to blood perfusion. For example, the mammalian embryo develops in a low-O_2_ environment, and in this context, hypoxia-inducible factor (HIF) has additional responsibilities in multiple processes (Dunwoodie, 2009). In vertebrate models, the developing LB expresses HIF2A prior to the initiation of fetal circulation (Lin et al., 2014), indicating that early hepatoblast expansion and delamination require transient exposure to hypoxic conditions. Similarly, in the developing kidney, both HIFA isoforms are activated in a cell-specific and temporally controlled manner, indicating a regulatory role for O_2_ tension in nephrogenesis (Bernhardt et al., 2006). These reports suggest that physiological hypoxia potentially impacts early organ development.

The aim of this study was to evaluate the role of O_2_ conditions in directing liver organoids and early liver development. Specifically, to clarify the precise hypoxic role in liver development, we characterized the influence of variable O_2_ tensions with O_2_-permeable culture plates using hiPSC-LBs as a fetal liver model.

## Results

### Hypoxic Conditions Generated with an O_2_-Permeable Plate Promoted Hepatocyte Differentiation in Liver Buds

**Figure 1:**
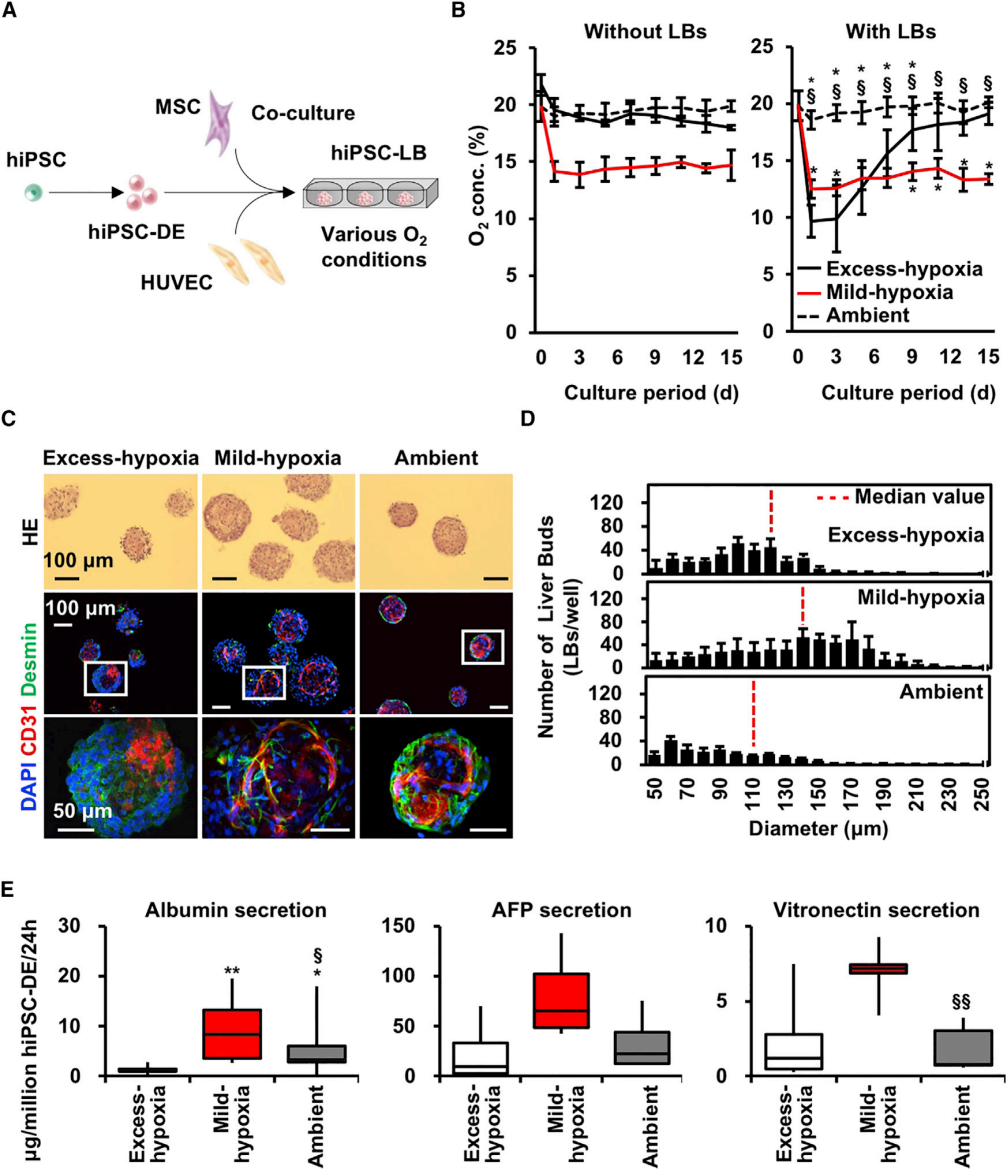
Hypoxic Conditions Generated with an O_2_-Permeable Plate Promoted Hepatocyte Differentiation in Liver Buds (A) Schematic view of the present study. (B) Changes in O_2_ tension in culture medium without (left) or with (right) hiPSC-LBs for 15 days (mean ± SD; n = 8 or 9 independent experiments; ^∗^p < 0.05 versus Excess-hypoxia; ^§^p < 0.05 versus Mild-hypoxia). (C) H&E and immunofluorescence staining of hiPSC-LBs cultured for 15 days (CD31: red; Desmin: green; nuclei: blue [DAPI]). Scale bar, 100 μm (upper and middle side) or 50 μm (lower side). (D) Diameter distribution of hiPSC-LBs cultured for 15 days (mean ± SD; n = 9–11 independent experiments). (E) Boxplots of hiPSC-DE cell number normalized protein level in hiPSC-LB on day15. The error bars represent the maximum and minimum values; n = 8–18 independent experiments; ^∗^p < 0.05 and ^∗∗^p < 0.01 versus Excess-hypoxia; ^§^p < 0.05 and ^§§^p < 0.01 versus Mild-hypoxia.

As the use of O_2_-impermeable culture plates that applied the strong hypoxic environment, a polydimethylsiloxane (PDMS) plate was used due to its stability and tunability of O_2_ conditions (Figures 1A, 1B, and S1A). All of the hypoxic culture conditions assessed in this study are summarized in Figure S1A.The most important feature of this plate is that it provides a direct O_2_ supply to the cells because the plate is made of PDMS that is only permeable to gas (Anada et al., 2012), promoting the differentiation and survival of 3-D cultured MSCs (Anada et al., 2016, Kamoya et al., 2016). Conversely, a lower O_2_ level incurred using polystyrene plates with low O_2_ permeability, because PDMS's O_2_ permeability limits the ability to control low O_2_ tension (Byrne et al., 2014). Excess-hypoxia condition enables a comparison between test sets. Mild-hypoxia (10% O_2_, O_2_-permeable plate) and Ambient (20% O_2_, O_2_-permeable plate) condition equilibrate culture medium with external O_2_ tension, with minimum values of 13% and 19%, respectively (Figure 1B). However, dissolved O_2_ modulated with Excess-hypoxia condition (20% O_2_, O_2_-non-permeable plate) had the lowest (10%) O_2_ levels under all conditions during the first 3 to 4 days and then the O_2_ tension gradually increased. Although the CO_2_ level in the medium of Ambient condition was slightly low, the pH value in all groups was stable at approximately pH 7 (Figure S1B). Thus, the PDMS plate is adequate for evaluation of higher O_2_ conditions.

To induce hiPSC-LB self-organization, hiPSC-derived definitive endoderm cells (hiPSC-DE) were mixed with HUVECs and MSCs (Figure 1A). Then, the cell mixture was seeded onto micro-patterned plates and cultured under various O_2_ conditions. After 15 days of culture, HUVECs in hiPSC-LBs cultured on Mild-hypoxia and Ambient condition formed a network structure, and MSCs were observed to be located adjacent to HUVECs (Figure 1C). Moreover, hiPSC-LBs cultured on Mild-hypoxia condition showed the highest median diameter (Figure 1D). In addition, these hiPSC-LBs exhibited the highest average LB number per well and the highest diameter ratio (over 150 μm) (Figures S1C and S1D).

In order to extrapolate the ratio of hiPSC-DE and stromal cells (HUVECs and MSCs) in hiPSC-LB cultured for 15 days, we counted CK8/18 stained cells divided by DAPI-stained nuclei to estimate hiPSC-DE cell percentage (Figure S1E). Although the initial ratio of hiPSC-DE and stromal cells were 1 and 0.9 in hiPSC-LB, the eventual ratio of hiPSC-DE and stromal cell in hiPSC-LBs was 0.91 versus 0.09 in Excess-hypoxia, 0.66 versus 0.34 in Mild-hypoxia and 0.83 versus 0.17 in Ambient on day 15 in immunohistochemistry of respective lineage makers (Figure S1E). Combined with total genomic DNA amount (Figure S2E), we estimate hepatic cell numbers in a well that were 1.5 × 10^5^, 1.8 × 10^5^, and 1.5 × 10^5^ in Excess-hypoxia, Mild-hypoxia, and Ambient, which are approximately 30% of seeding time and calculated based on a published literature, which estimates from the genomic DNA amount in a cell at 6.57 pg/cell (Figure S2F). Given that almost all hiPSC-LBs were collected (we established collection rate of >98% as criterion), the reduction of hiPSC-DE suggests presence of cell death in hiPSC-LB associated with long-term culture. Additionally, the stromal cell numbers in a well were 0.14 × 10^5^, 0.93 × 10^5^, and 0.32 × 10^5^ in Excess-hypoxia, Mild-hypoxia, and Ambient, although the number was 4.3 × 10^5^ at the seeding time. Therefore, the hypoxic culture in Mild-hypoxia group most efficiently preserved stromal lineage in hiPSC-LB.

Regarding average albumin secretion value normalized by the number of hiPSC-DE cells in a well, the Mild-hypoxia group (8.6 μg/million hiPSC-DE/24 hr) was approximately six times higher than the Excess-hypoxia group (1.4 μg/million hiPSC-DE/24 hr) and approximately two times higher than the Ambient group (4.1 μg/million hiPSC-DE/24 hr) on day 15 (Figure 1E). The secretion of vitronectin (a cell adhesion protein) in the Mild-hypoxia group (6.9 μg/million hiPSC-DE/24 hr) was also significantly higher than the Ambient group (1.7 μg/million hiPSC-DE/24 hr), although the secretion of alpha-fetoprotein (AFP; a fetal liver marker) was not significant. LBs cultured on Mild-hypoxia condition also demonstrated an increase in the basal level of cytochrome P450 family 3 subfamily A member 4 (CYP3A4) activity and urea production (Figures S2A and S2B). Additionally, immunofluorescence indicated that all the liver markers examined were expressed in all groups (Figure S2C). *ALB* and *RBP4* gene expression levels normalized to 18s rRNA expression of hiPSC-DE in hiPSC-LB were higher in the LBs cultured on Mild-hypoxia and Ambient condition than those in the LBs grown on Excess-hypoxia condition (Figure S2D). These data suggest that Mild-hypoxia promotes human hepatoblast differentiation in hiPSC-LBs rather than just a proliferation.

### TGFB Signals from the Mesenchyme and Endothelium Are Candidate Regulators of O_2_-Dependent Hepatocyte Differentiation in LBs

In embryonic day 13.5 (E13.5) mice, the hepatoblasts starts to differentiate into either hepatocytes or cholangiocytes (Zorn, 2008), and TGFBs and JAGGED1 (JAG1) direct the fate of these cells by promoting hepatoblast differentiation into cholangiocytes (Zorn, 2008, Gordillo et al., 2015). TGFB inhibits the proliferation of both hepatocytes and endothelial cells (ECs) (Nguyen et al., 2007, Takehara et al., 1987) and hepatocyte differentiation (Spagnoli et al., 2000). Additionally, TGFB induces apoptosis in both hepatocytes and ECs (Black et al., 2007, Ferrari et al., 2012). Consistently, our fetal mouse liver organ culture indicated that TGFB3 inhibits liver outgrowth, and TGFB3 signaling inhibition suppresses *Sox9* (cholangiocyte marker) gene expression (Figures S3A–S3C). Given that early hepatic organogenesis is accompanied by TGFB modulation across multiple cell types, we hypothesized that Mild-hypoxia condition-induced hepatocyte differentiation occurred in hiPSC-LBs by means of TGFB reduction.

**Figure 2:**
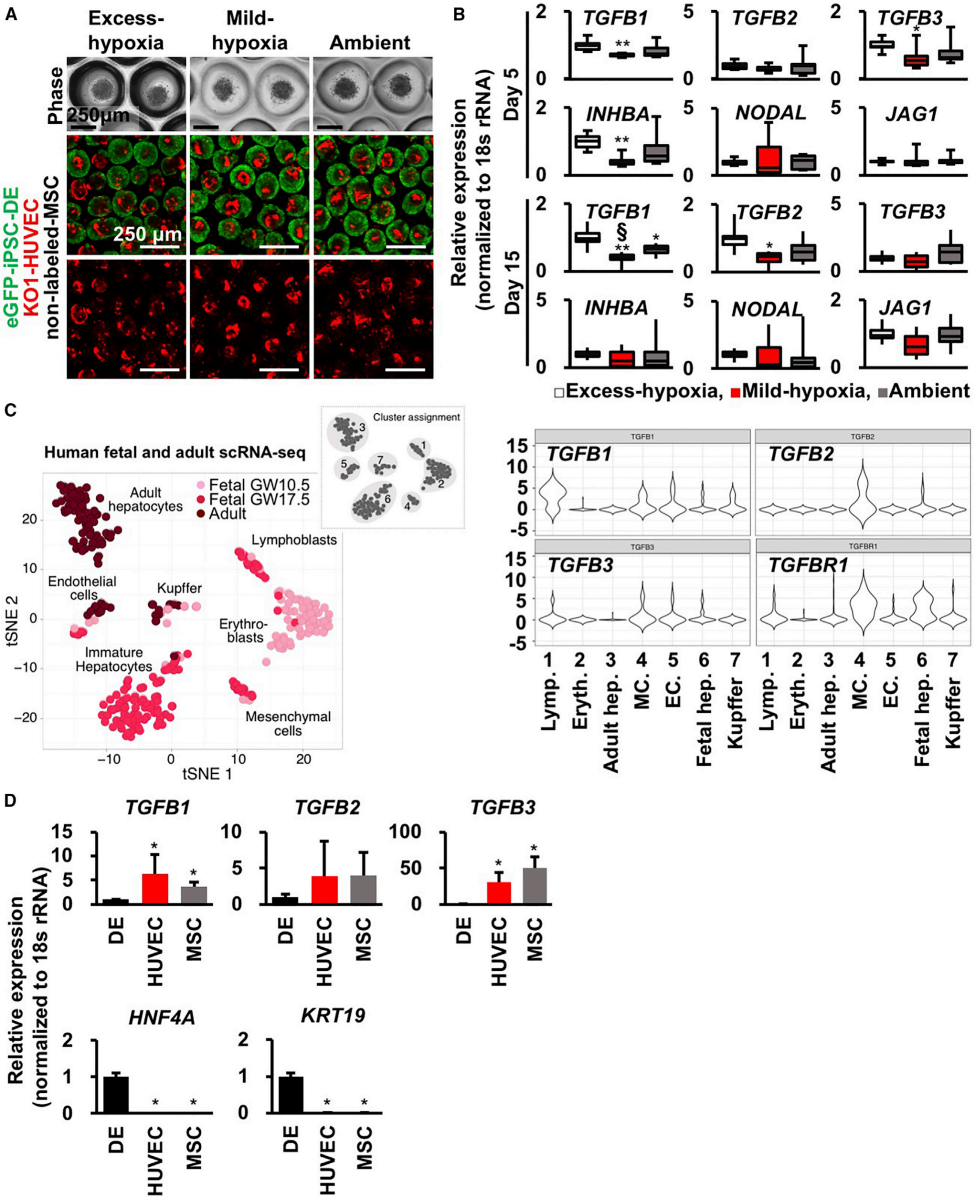
TGFB Signals from the Mesenchyme and Endothelium Are Candidate Regulators of O_2_-Dependent Hepatocyte Differentiation in Liver Buds (A) Phase-contrast and confocal images of hiPSC-LBs cultured for 1 (phase) or 5 (confocal) days (green: eGFP-iPSC-DE cells [AAVS1:EGFP]; red: KO1-HUVECs [MSCV-KO1]; no label: MSCs; scale bar, 250 μm). (B) Boxplots of TGFB family gene expression in hiPSC-LBs cultured for 5 and 15 days. The error bars represent the maximum and minimum values; n = 9 (day 5) and 10 (day 15) independent experiments; ^∗^p < 0.05 and ^∗∗^p < 0.01 versus Excess-hypoxia; ^§^p < 0.05 versus Ambient. (C) Left: tSNE of single-cell transcriptomes from human fetal (two donors, gestation weeks 10.5 and 17.5, 238 cells) and adult (three donors, age 21–65, 256 cells) liver samples (modified from Camp et al., 2017). Right: violin plots of single-cell transcriptomes, showing the distribution in the human liver. (D) Gene expression of each cell lineage in hiPSC-LB. hiPSC-LBs were cultured on Ambient group for 2 days. Then, dissociated eGFP-DE cells, KO-HUVECs, and unlabeled-MSCs from hiPSC-LBs were separated by FACS analysis using fluorescence labels. Gene expression of these cells was analyzed (mean ± SD; n = 8 independent experiments; ^∗^p < 0.05 versus hiPSC-DE cells).

To analyze the role of variable O_2_ condition on TGFBs and JAG1, we quantified gene expression in the hiPSC-LBs on days 5 and 15 (Figures 2A and 2B). The TGFB1, TGFB3, and inhibin beta A subunit (INHBA) gene expression levels in hiPSC-LBs grown on Mild-hypoxia was significantly lower than those in hiPSC-LBs cultured on Excess-hypoxia (*TGFB1*, 0.7; *TGFB3*, 0.6; *INHBA*, 0.4 versus Excess-hypoxia; mean of gene expression) on day 5. Moreover, the TGFB1 gene expression levels in hiPSC-LB cultured on Mild-hypoxia was significantly lower than the Ambient group on day 15 (0.4; versus Ambient; mean of gene expression). Additionally, the O_2_ concentration in Mild-hypoxia group is also significantly lower than Ambient group on day 15. Therefore, the Mild-hypoxia condition is the most efficient in reducing TGFB expression and provides a favorable environment for hepatocytic differentiation.

To determine which cells produce TGFBs in the human liver, we reanalyzed the gene expression of human liver using single-cell RNA sequencing (Camp et al., 2017) (Figure 2C). The *TGFB2* gene was clearly expressed in mesenchymal cells (MCs) in the liver, and both *TGFB1* and *TGFB3* were expressed in ECs and MCs. Moreover, TGFB receptor 1 (TGFBR1) was clearly expressed in fetal hepatocytes and MCs. We then performed fluorescence-activated cell sorting (FACS) using dissociated hiPSC-LBs to investigate the cellular source for TGFB ligands (Figure 2D). Interestingly, *TGFB1* and *TGFB3* gene expression levels were significantly higher in isolated HUVECs and MSCs than those in hiPSC-DE cells. In addition, hepatocyte nuclear factor 4 alpha (*HNF4A*; hepatocyte marker) and cytokeratin 19 (*KRT19*; cholangiocyte marker) expression levels were higher in hiPSC-DE cells than those in other cells. These results indicate that HUVECs and MSCs produce major TGFB1 and 3 in hiPSC-LBs. This suggests that the major source of TGFB supply in hiPSC-LBs seems consistent with that in human fetal liver cells. Given that the signal from the portal MCs and/or portal vein ECs is a candidate promoter of hepatoblast maturation toward cholangiocytes (Clotman et al., 2005), balanced TGFB1 and 3 signals from MCs and ECs are likely regulators of O_2_-dependent hepatocyte differentiation in the LB.

### *Hif1a* Is Positively Correlated with *Tgfbs* and Biliary Markers and Negatively Correlated with Hepatocyte Markers in Mouse Liver Development

**Figure 3:**
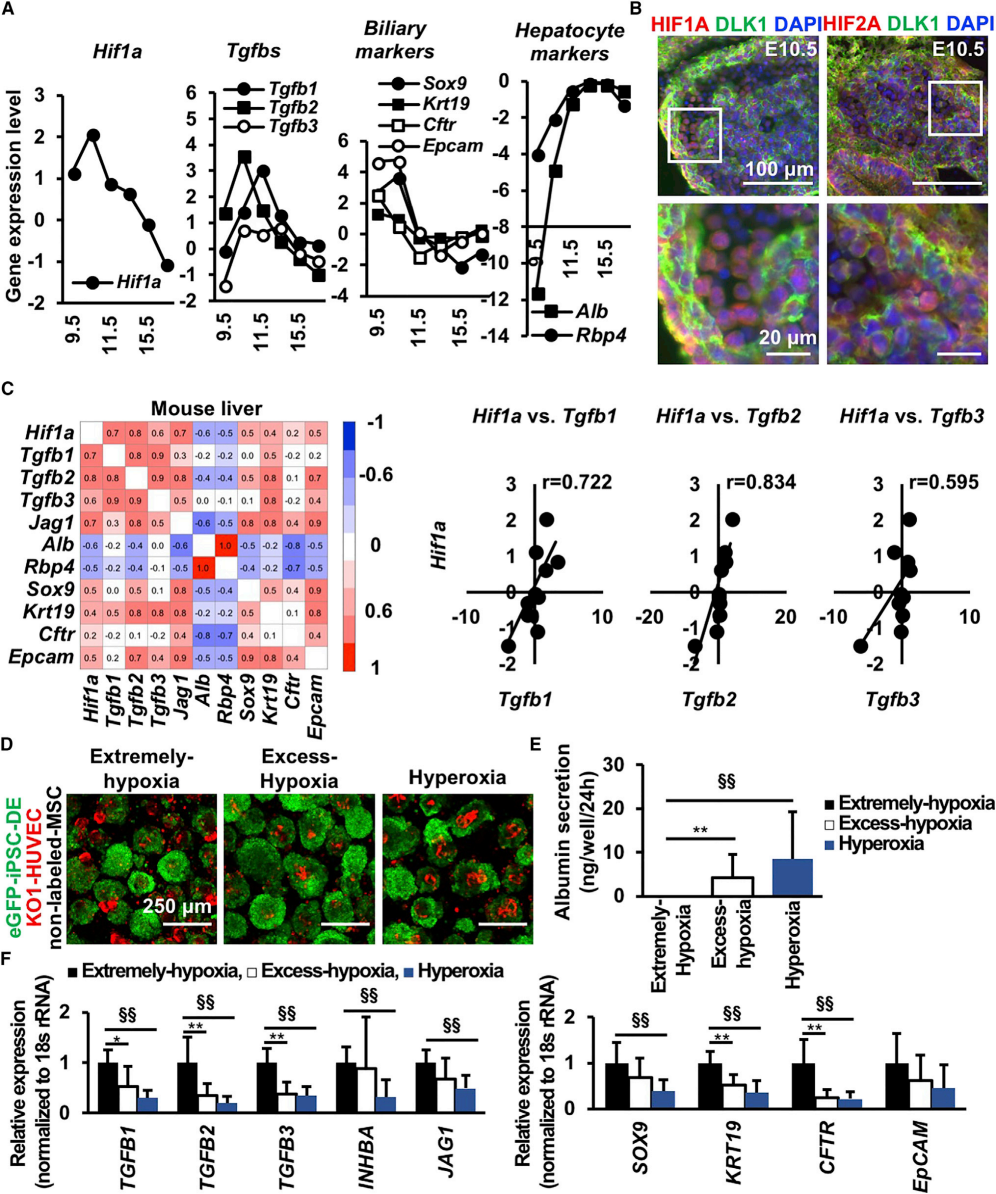
*Hif1a* Is Positively Correlated with *Tgfbs* and Biliary Markers and Negatively Correlated with Hepatocyte Markers in Mouse Liver Development (A) Microarray gene expression analysis of mouse fetal liver from E9.5 to 17.5. (B) Immunofluorescence staining for HIF1A (red), HIF2A (red), DLK1 (green), and nuclei (blue, DAPI) in E10.5 mouse liver. Scale bar, 100 μm (upper) or 20 μm (lower). (C) Correlation analysis of hypoxia- (*Hif1a*), hepatocyte- (*Alb* and *Rbp4*), and cholangiocyte- (others) associated markers in mouse livers from E9.5 to 8-week-old mice. (D) hiPSC-LBs cultured for 10 days (green: eGFP-iPSC-DE cells [AAVS1:EGFP]; red: KO1-HUVECs [MSCV-KO1]; no label: MSCs; scale bar, 250 μm). (E) ELISA on protein secretion in hiPSC-LBs cultured for 10 days (mean ± SD; n = 15 independent experiments; ^∗∗^p < 0.01 versus Excess-hypoxia, ^§§^p < 0.01 versus Extreme-hypoxia). (F) Gene expression in hiPSC-LBs cultured for 10 days (mean ± SD; n = 12 independent experiments; ^∗^p < 0.05 and ^∗∗^p < 0.01 versus Excess-hypoxia, ^§§^p < 0.01 versus Extreme-hypoxia).

To investigate whether O_2_-dependent LB development is present *in vivo*, we compared mouse fetal liver microarray analyses on multiple embryonic days (Figure 3A). The mouse LB is established before E10 (Zorn, 2008, Gordillo et al., 2015), and blood circulation is initiated and sophisticated after LB generation (Swartley et al., 2016). Therefore, *Hif1a* expression is hypothetically influenced by O_2_ tension change through fetal circulation development. During early hepatogenesis, along with the gradual reduction of *Tgfb* gene expression, cholangiocyte markers of SRY ([sex determining region Y]-box 9 [*Sox9*]) and cystic fibrosis transmembrane conductance regulator (*Cftr*) decreased, whereas hepatocyte markers, such as *Alb* and *Rbp4*, increased over time. Immunofluorescence indicated the presence of HIF1A and HIF2A expression in E10.5 fetal livers (Figure 3B). These data indicate that mouse LB development is initiated under potent hypoxic conditions and hepatic gene expression is increased after liver circulation develops. Correlation analysis indicated that *Hif1a*; *Tgfb*1, 2, and 3; and biliary markers were positively correlated, and *Hif1a* and hepatocyte markers were negatively correlated (Figure 3C). Thus, hepatic and biliary gene expressions were influenced by hypoxic signatures *in vivo*.

Considering the potential O_2_-dependent TGFB modulation in the *in vitro* LB model, the effect of stronger hypoxic conditions in LB differentiation was investigated (Figures 3D and S3E). Compared with the hiPSC-LBs cultured under Excess-hypoxia and/or hyperoxia conditions, those cultured under Extreme-hypoxia conditions exhibited downregulated albumin secretion (Figure 3E) and upregulated *TGFB* family genes and cholangiocyte marker gene expression (Figure 3F). Immunofluorescence also indicated that, compared with Strong-hypoxia and Mild-hypoxia condition, hypoxic conditions increase cholangiocyte marker expression and decrease hepatocyte marker expression (Figures S3F and S3G). Additionally, correlation analysis of *HIF1A*, *TGFB*, biliary marker, and hepatocyte marker gene expression in hiPSC-LBs indicated a pattern similar to that in the mouse liver (Figures 3C and S4A). These data suggest the appropriate hypoxic condition permits hepatoblast lineage commitment through the modulation of TGFB signaling.

To investigate whether MSCs and HUVECs in hiPSC-LB is necessary for influencing hepatoblast differentiation through TGFB and O_2_ tension, we investigated hepatic function of 3D cultured hiPSC-DE cells. Albumin secretion is two times lower by hiPSC-DE spheroids than hiPSC-LB in Mild-hypoxia condition (Figure S4B), suggesting the presence of stromal cells significantly augments hepatic differentiation in hypoxic condition. Hepatic differentiation again measured by albumin levels in Extreme-hypoxia and Excess-hypoxia group are significantly lower than in the Mild-hypoxia group (Figure S4C). In agreement with a previous study (Hung et al., 2013), the expression level of TGFB1 at day 5 in Excess-hypoxia is significantly higher than Mild-hypoxia (Figure 2B). A previous literature reported that the half-life of TGFB1 mRNA is 15 hr (Bascom et al., 1989). Therefore, TGFB1 gene expression level at day 5 is likely affected by the day 3 to 4 condition.

To highlight the cell-type-specific mechanism for promoting hepatic differentiation, we interfered TGFB1 gene in stromal lineages (HUVEC or MSC) of hiPSC-LB in Excess-hypoxia condition (Figure S4D). Gene expression of *ALB* and *TTR* in TGFB1 knocked down hiPSC-LB is approximately 1.5 to 2.5 times higher than control in Excess-hypoxia condition at day 4. On day 15, TGFB1 gene expression and O_2_ concentration in the Ambient group is higher than in the Mild-hypoxia group, and albumin secretion level in the Ambient group is lower than in the Mild-hypoxia group (Figures 2B, 1B, and 1E). Thus, upregulated TGFB1 expression level relative to the Mild-hypoxia condition likely impairs hepatic differentiation. These data suggest that optimal hypoxia regulates hiPSC-LB differentiation through intercellular TGFB signaling.

### TGFB Signal Inhibition Promotes Hepatocyte Differentiation in LBs

**Figure 4:**
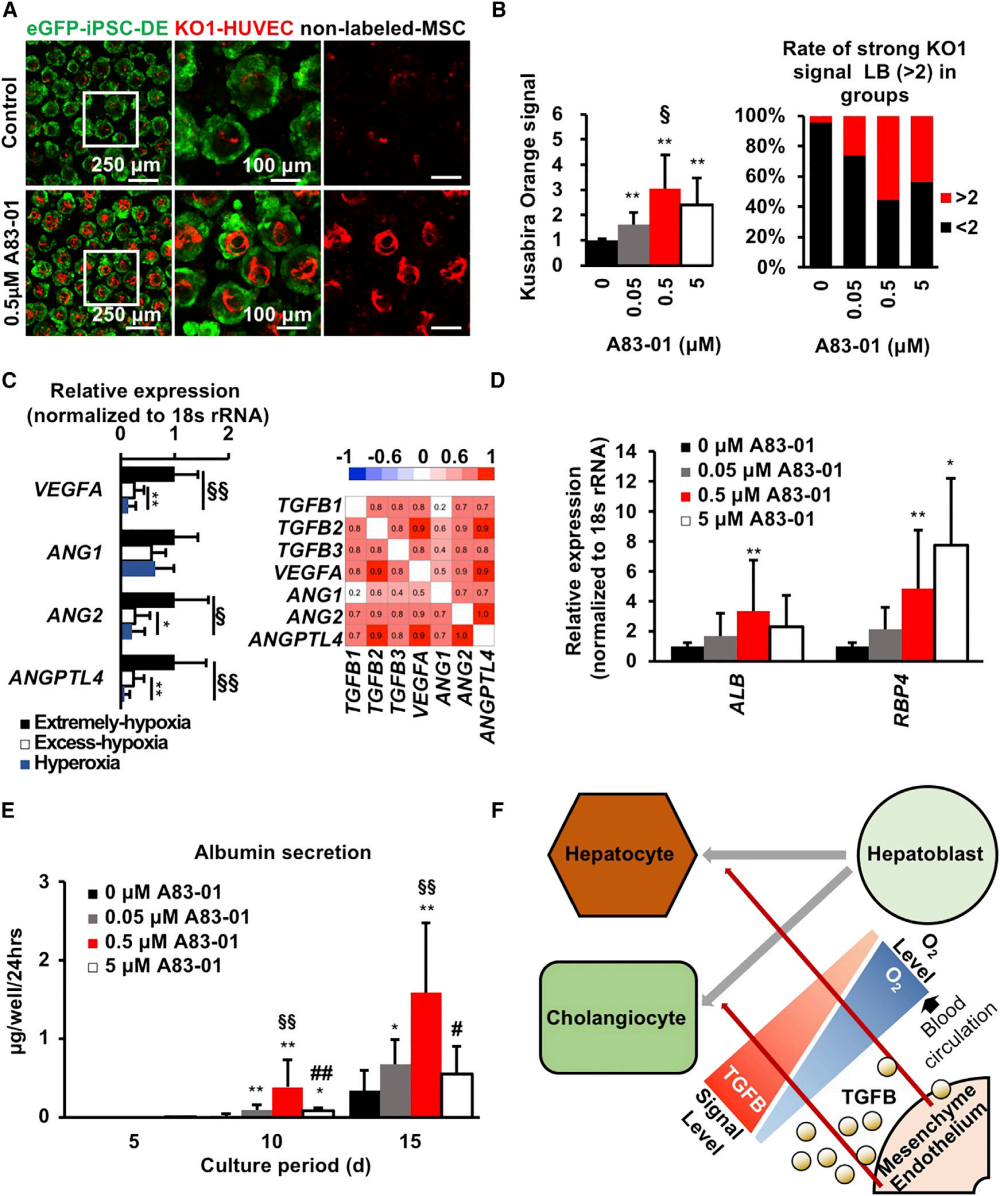
TGFB Signal Inhibition Promotes Hepatocyte Differentiation in Liver Buds (A) Confocal imaging of hiPSC-LBs cultured with various concentrations of A83-01 for 15 days in Excess-hypoxia group (green: eGFP-iPSC-DE cells [AAVS1:EGFP]; red: KO1-HUVECs [MSCV-KO1]; no label: MSCs; scale bar from left to right, 250, 100, and 100 μm). (B) Image analysis of HUVEC abundance in hiPSC-LBs cultured with various A83-01 concentrations for 15 days in Excess-hypoxia group. Fluorescence intensity of KO1 protein expression in HUVECs was evaluated as HUVEC abundance in hiPSC-LBs (left: mean ± SD; n = 9–17 independent experiments; ^∗∗^p < 0.01 versus 0 μM; ^§^p < 0.05 versus 0.05 μM; right: a total of 1449–2985 LBs were measured). (C) Gene expression and correlation of hiPSC-LBs cultured for 10 days (mean ± SD; n = 12 independent experiments; ^∗^p < 0.05 and ^∗∗^p < 0.01 versus Excess-hypoxia, ^§^p < 0.05 and ^§§^p < 0.01 versus Extreme-hypoxia). (D) Gene expression in hiPSC-LBs cultured with various A83-01 concentrations for 15 days in Excess-hypoxia group (mean ± SD; n = 6–14 independent experiments; ^∗^p < 0.05 and ^∗∗^p < 0.01 versus 0 μM). (E) ELISA of protein secretion in hiPSC-LBs cultured with various A83-01 concentrations for 15 days in Excess-hypoxia group (mean ± SD; n = 9–17 independent experiments; ^∗^p < 0.05 and ^∗∗^p < 0.01 versus 0 μM; ^§§^p < 0.01 versus 0.05 μM, ^#^p < 0.05 versus 0.5 μM; ^##^p < 0.05 versus 0.5 μM). (F) Putative mechanism of O_2_-dependent differentiation in human LBs through TGFB signaling.

To investigate the effects of TGFB on hiPSC-LBs, we blocked TGFB signaling using the TGFB inhibitor A83-01 in the Excess-hypoxia group (Figures 4A and 4B). The hiPSC-LBs were collected on day 15 and Kusabira-Orange fluorescent protein (KO1) fluorescence intensity was measured to quantify the abundance of KO1-labeled HUVECs. As expected, blocking TGFB signaling gradually increased the abundance of HUVECs at an A83-01 concentration of up to 0.5 μM, and the HUVEC abundance was three times higher than that in the control. The rate of hiPSC-LBs, which exhibit high KO1 fluorescence intensity, was 5% in 0 μM A83-01 and 55% in 0.5 μM A83-01. The expression level of angiogenesis-related cytokines, such as vascular endothelial growth factor A (*VEGFA*), angiopoietin2 (*ANG2*), and angiopoietin-like 4 *(ANGPTL4*) (Figure 4C) increased under hypoxic conditions and was positively correlated with that of *TGFB*s. TGFBs induce EC apoptosis mediated by VEGF signaling (Ferrari et al., 2012); ANG2 antagonizes the vascular receptor tyrosine kinase (Augustin et al., 2009), and ANGPTL4 inhibits EC adhesion, migration, and sprouting (Cazes et al., 2006). These data suggest that O_2_ tension and TGFBs influence angiogenesis in hiPSC-LBs. In addition, gene expression and protein secretion of hepatocyte makers in hiPSC-LBs in the Excess-hypoxia group gradually increased in a dose-dependent manner (Figures 4D and 4E). On day 15, *ALB* gene expression was the highest in 0.5 μM A83-01-treated LBs and *RBP4* expression was highest in 5 μM A83-01-treated LBs. Albumin secretion was also increased with concentrations up to 0.5 μM A83-01, and the value was 1.5 μg/well/24 hr, which was five times higher than that in the control. However, 5 μM A83-01 induced a significant reduction in albumin secretion and HUVEC abundance as well as a reduction in *ALB* gene expression. Finally, we compared the albumin levels in hiPSC-LBs with those in frozen adult hepatocytes (Figure S4F). Albumin secretion and gene expression levels in hiPSC-LBs on the Mild-hypoxia condition was 10-fold higher than those of frozen adult hepatocytes. These data suggest that appropriate TGFB signals derived from HUVECs and MSCs promote hepatocyte differentiation in LBs. These data support a potential mechanism of O_2_-dependent differentiation in human LBs through TGFB signaling (Figure 4F).

## Discussion

All three TGFB isoforms (TGFB1, 2, and 3) are known to induce biliary marker expression and repress hepatocyte marker expression in mouse hepatoblasts (Antoniou et al., 2009). *Tgfb1* mRNA was expressed widely in the fetal mouse liver, whereas *Tgfb2* and *Tgfb3* mRNA was found predominantly in the periportal region (Antoniou et al., 2009). In the present study, *Tgfb1* expression in fetal mouse liver decreased in a time-dependent manner from the initiation of blood circulation with a positive correlation to the hypoxic response. Therefore, this *Tgfb1* reduction stimulates differentiation of hepatoblasts into hepatocytes rather than cholangiocytes coincided by fetal circulatory system establishment.

Additionally, excess TGFB inhibition impaired hiPSC-LB differentiation, suggesting optimal TGFB expression in HUVECs and MSCs is important for hepatocyte differentiation. Although the reason is uncertain, TGFB also has a positive effect on fetal hepatocyte development. A previous study showed that TGFB upregulates fibronectin expression in mouse embryos (Sugiyama et al., 2013), and fibronectin upregulates hepatic gene expression in rat fetal hepatocytes (Sanchez et al., 2000).

During liver regeneration, the TGFB family cytokine activin A works as a negative regulator of hepatocyte proliferation and serves as a candidate terminator of liver regeneration (Bohm et al., 2010). We previously reported that iPSC-DE cells co-cultured with HUVECs or MSCs using a 2D culture system exhibit decreased albumin production and hepatic maturation (Asai et al., 2017). Hypoxic factors, including INHBA (an activin subunit), are candidate factors for explaining this discrepancy. Mild-hypoxia (PDMS plate in 10% O_2_) condition reduced the gene expression of *INHBA* and other *TGFB* family members. Moreover, the activin A signal is a target of A83-01. Although further mechanistic investigation of other possible factors linking hypoxia with hepatic differentiation in developing liver will be needed, optimized O_2_ conditions together with multicellular co-culture will be a potential strategy to promote maturation into the hepatocytic lineage.

Collectively, timely control of hypoxia induction to hiPSC-LB or mouse LB promoted the stable hepatocytic differentiation. However, it remains unclear how the spatial gradient of developmental hypoxia contributes early differentiation in LB. Toward this goal, combinatorial use of a tissue engineering approach will be promising for enabling *in vitro* optimized O_2_ delivery such as through a perfusion device. Future efforts to optimize spatiotemporal O_2_ control will produce functional and reproducible liver organoids, which will be advantageous for regenerative medicine and drug screening applications.

## Experimental Procedures

Experimental procedures are provided in the Supplemental Information.

### Cell Culture, DE Differentiation, and Tissue Co-culture

Donors' informed consents were obtained for all experiments using human tissue, and all procedures involving the use of human cells and mice were approved by the ethics commission of Yokohama City University. In the absence of any mention, all cells were maintained at 37°C in a humidified atmosphere containing 5% CO_2_ and 95% ambient air. The hiPSC line, TkDA3-4 (kindly provided by Dr. Nakauchi at University of Tokyo), was maintained on Laminin 511 E8 fragment (iMatrix-511, kindly provided by Nippi)-coated dishes in StemFit (Ajinomoto). The EGFP knocked-in to adeno-associated virus integration site 1 (AAVS1:EGFP) was used for live imaging analysis *in vitro*. The differentiation protocol for the induction of hiPSC-DEs was based on a previous report with some modifications (Kajiwara et al., 2012). Briefly, hiPSCs were seeded on a Laminin 511 E8-fragment-coated (iMatrix-511, kindly provided by Nippi) dish with RPMI-1640 (Wako) with 1% B-27 Supplement, serum free (Gibco) containing 100 ng/mL activin A (kindly provided by Ajinomoto) and 50 ng/mL Wnt3a (R&D Systems). The medium was used for 6 days. At cell seeding (D0), 10 μM ROCK Inhibitor Y-27632 (Wako) was added, and 1 mM sodium butyrate (Sigma) was added from D1 to D3. HUVECs and MSCs were cultured in endothelial cell growth medium (EGM; Lonza) or MSC growth medium (MSCGM; Lonza). For live imaging experiments, HUVECs were fluorescently labeled with KO1-encoding retroviruses constructed from a retrovirus vector pGCDNsam possessing a long terminal repeat derived from murine stem cell virus (MSCV). In brief, 293-gp and 293-gpg packaging cells were transfected with the retrovirus vector pGCDNsam-IRES KOFP (kindly provided by Dr Masafumi Onodera) and the culture supernatants were used for infection (Camp et al., 2017). Viable human adult hepatocytes (BioreclamationIVT; lots IVT-M00995) were cultured in the modified Lanford medium (Nissui) and albumin secretion were measured on the first 24 hr of culture. The mouse livers collected from E13.5 fetal mice were cut into block and culture for 72 hr onto 6.5-mm-diameter Transwell polycarbonate filter membranes (0.4 μm pore size; Corning) in a 24-wells plate holding 250 μL/well of medium under 10% O_2_. The medium consisted of DMEM (high glucose) with L-glutamine and phenol red (Wako) containing 10% fetal bovine serum (biowest), 5% Matrigel (Corning), and 100 units/mL penicillin-streptomycin (Gibco). A Leica TCS SP8 confocal microscope (Leica Microsystems) was used for imaging analysis of fetal mouse liver. z axis height was equalized for comparison of each group. ImageJ software (NIH) was used to quantify.

### Generation of Human iPSC-LBs

To generate hiPSC-LBs, we used micro-patterned plates made from O_2_-permeable PDMS (Anada et al., 2012) or little permeable polystyrene. Total 9.0 × 10^5^ cells of hiPSC-DE, HUVEC, and MSC were co-cultured on a well at the ratio of 10:7:2 in hepatocyte culture medium (HCM)/EGM medium. The medium is a 1:1 mixture of HCM (Lonza) without epidermal growth factor and EGM (Lonza) containing dexamethasone (0.1 mM, Sigma-Aldrich), oncostatin M (10 ng/mL, R&D Systems), hepatocyte growth factor (20 ng/mL, Kringle Pharma) and 2.5% fetal bovine serum, CELLect GOLD (MP Biomedicals). Only seeding time, 10 μM ROCK Inhibitor was added in the medium. To investigate the effect of O_2_, hiPSC-LBs were cultured in 2%, 10%, 20%, and 40% O_2_ during research periods. In TGFB signal inhibition research, 0 to 5 μM A83-01 (Wako) was added into culture medium for 15 days and 1 μM ROCK Inhibitor on the initial day. The diameters of collected hiPSC-LBs were measured by the IN-Cell Analyzer 2000 system (GE Healthcare). A Leica TCS SP8 confocal microscope (Leica Microsystems) was used for imaging analysis of hiPSC-LBs. z axis height and laser power were equalized for comparison of each groups. ImageJ software (NIH) was used to quantify average intensity of Kusabira-Orange fluorescence in hiPSC-LBs.

### Microarray and scRNAseq

Gene expression data are downloaded and reanalyzed from the publicly available GEO, NCBI under accession numbers GEO: GSE46631, GSE81252, and GSE96981.

## Author Contributions

H.A. conceived the study, designed and performed the experiments, collected and analyzed the data, and wrote the manuscript. T.A., T.K., T.S., M.K., E.Y., S.K., A.F., Y.U., K.S., J.G.C., and B.T. performed and analyzed the experiments. T.T. conceived the study, designed the experiments, wrote and reviewed the manuscript, optimized the experiments, and supervised the study. O.S. and H.T. supervised the study.

## Supplemental Information

Document S1. Supplemental Experimental Procedures and Figures S1–S4

Document S2. Article plus Supplemental Information
