# Supplementary material for: Optimal Hypoxia Regulates Human iPSC-Derived Liver Bud Differentiation through Intercellular TGFB Signaling
Source: Stem Cell Reports. 2018 Jul 19;11(2):306–16. doi: 10.1016/j.stemcr.2018.06.015 (PMC6092760; doi:10.1016/j.stemcr.2018.06.015)
Supplement: Document S1. Supplemental Experimental Procedures and Figures S1–S4 [file mmc1.pdf]

Stem Cell Reports, Volume *11*

Supplemental Information

Optimal Hypoxia Regulates Human iPSC-Derived Liver Bud Differentiation through Intercellular TGFB Signaling

Hiroaki Ayabe, Takahisa Anada, Takuo Kamoya, Tomoya Sato, Masaki Kimura, Emi Yoshizawa, Shunyuu Kikuchi, Yasuharu Ueno, Keisuke Sekine, J. Gray Camp, Barbara Treutlein, Autumn Ferguson, Osamu Suzuki, Takanori Takebe, and Hideki Taniguchi

SUPPLEMENTAL INVENTORY

Optimal Hypoxia Regulates Human iPSC-Derived Liver Bud Differentiation Through Intercellular TGFB Signaling

Hiroaki Ayabe[1], Takahisa Anada[2], Takuo Kamoya[2], Tomoya Sato[2], Masaki Kimura[4], Emi Yoshizawa[1], Shunyuu Kikuchi[1], Yasuharu Ueno[1], Keisuke Sekine[1], J. Gray Camp[3], Barbara Treutlein[3], Autumn Ferguson[4], Osamu Suzuki[2], Takanori Takebe[1,4,5,6], Hideki Taniguchi[1]

Inventory of supplemental Information

Figure S1: This figure is related to Figure 1, 2 3 and 4.

Figure S2: This figure is related to Figure 1 and 2.

Figure S3: This figure is related to Figure 2 and 3.

Figure S4: This figure is related to Figure 3 and 4.

Supplemental Information

Supplemental Figure and Legends

A

O_2 condition	Air O_2 (%)	O_2 level (%)	Plate O_2 permeability
Extremely-hypoxia	10	8	Low
Excess-hypoxia	20	10	Low
Mild-hypoxia	10	13	High
Ambient	20	19	High
Hyperoxia	40	NA	Low
Strong-hypoxia	2	NA	High

B

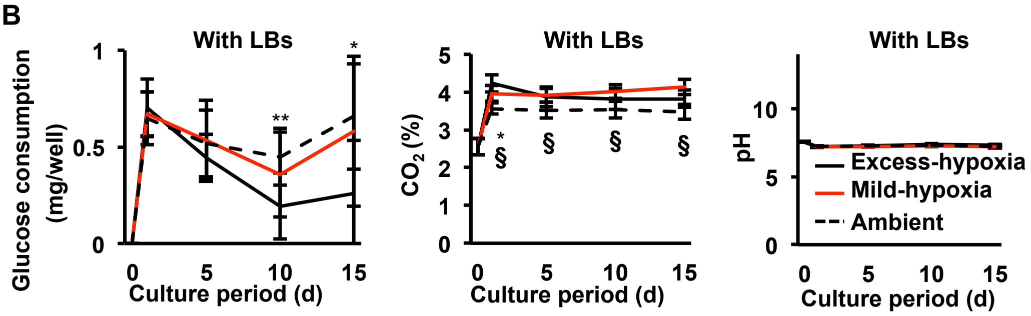

C

Test Group	n	Number (Liver buds/well)	Diameter (μm)	Median (μm)
Excess-hypoxia	11	353.5 ± 51.1	116.8 ± 8.9	122
Mild-hypoxia	9	489.3 ± 57.1]§§	139.3 ± 10.7]§§	143
Ambient	11	365.2 ± 96.8	110.4 ± 12.2	112

D

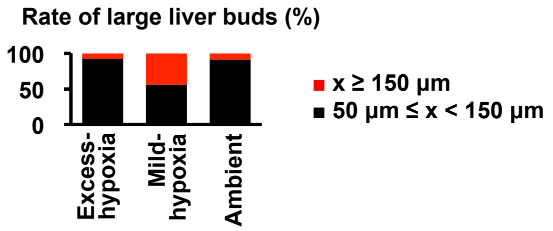

E

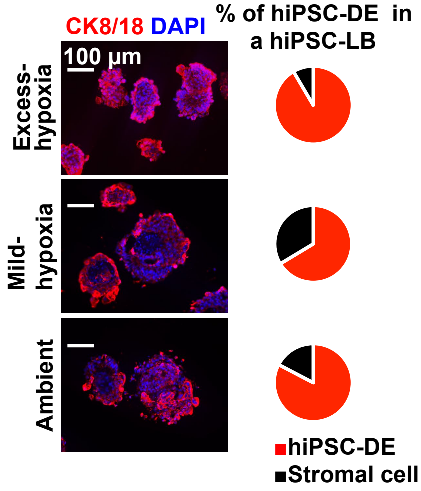

Figure S1. Test conditions of this study, environmental factor changes in hiPSC-LB culture on test set 1 and effect of O_2 in hiPSC-LB number/size. Related to Figure 1, 2, 3 and 4;

(A) Oxygen supply condition of hiPSC-LB cultured in this study. (B) Glucose consumption, CO_2 tension and pH change in culture medium with hiPSC-LB for 15 days (mean±SD; n=6~16 independent experiments; *: P<0.05 vs. Excess-hypoxia; §: P<0.05 vs. Mild-hypoxia). (C) Mean of hiPSC-LB number and diameter after 15 days culture (mean±SD; n=9~11 independent experiments; §§: P<0.01 vs. Mild-hypoxia). (D) Rate of over 150 μm hiPSC-LB in diameter after 15 days culture (From 3887 to 4404 hiPSC-LBs over 50 μm were tested). (E) Percentage of hiPSC-DE in a hiPSC-LB analyzed by immunohistochemistry on day15 (mean±SD; n=19~21; 4 independent experiments; CK8/18 (red) and Nuclei (DAPI; blue).

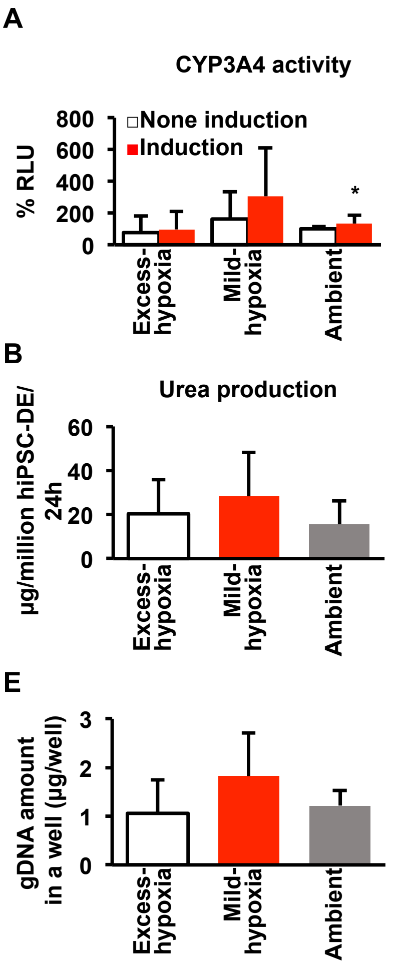

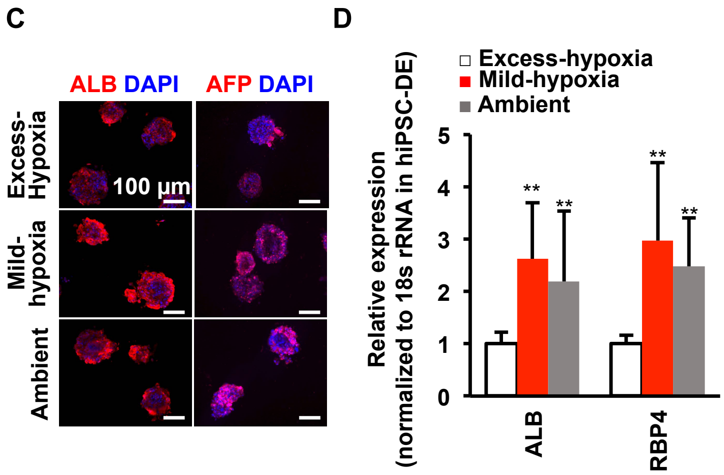

F

		Cells (x10E5)		
		hiPSC-DE	HUVEC, MSC	hiPSC-LB
Seeding Time	Day0	4.70	4.30	9.00
Excess-hypoxia	Day15	1.48±0.97	0.14±0.09	1.61±1.06
Mild-hypoxia	Day15	1.84±0.89	0.93±0.45	2.77±1.35
Ambient	Day15	1.53±0.39	0.32±0.08	1.85±0.48

Figure S2. Effect of O_2 in hiPSC-LB differentiation. Related to Figure 1 and 2;
(A) CYP3A4 activity in hiPSC-LB on day 20. Rifampicin was treated for 3 days. (mean±SD; n=11~13 independent experiments; *: P<0.05 vs. None induction). (B) Urea production in hiPSC-LB on day 20. NH_3Cl_4 was treated for 0 or 6 hours (mean±SD; n=11 independent experiments; **: P<0.01 vs. Excess-hypoxia). The values of urea production in a well were normalized by hiPSC-DE number in a well on day 15 (mean±SD; n=11 independent experiments). (C) IHC for ALB (red), AFP (red) and Nuclei (DAPI; blue) in hiPSC-LB on day15. Scale bar, 100 μm. (D) Relative gene expression in hiPSC-LB on day15. The gene expression levels were normalized to 18s rRNA expressed in hiPSC-DE. The ratio of each cell line was based on figure 2E (mean±SD; n=10 independent experiments; **: P<0.05 vs. Excess-hypoxia). (E) gDNA amount in a well on day15. mean±SD; n=10~11 independent experiments. (F) Cell number in a well on day 15 were calculated from hiPSC-DE and stroma (HUVEC and MSC) ratio in a hiPSC-LB Immunohistochemistry (IHC) and from gDNA amount in a well (6.57pg/cell; Serth et al., 2000).

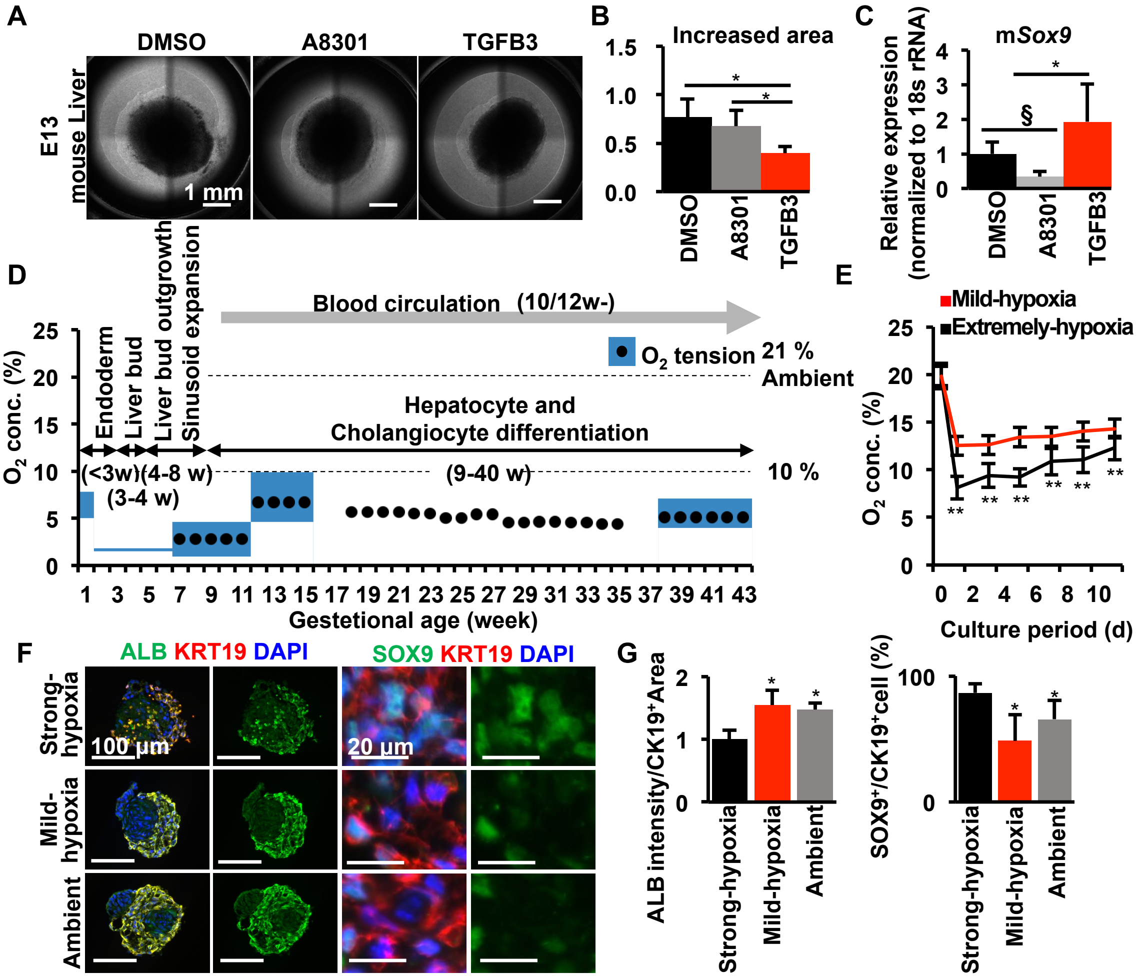

Figure S3. Effect of TGFB signal in fetal mouse liver, O_2 concentration change in human embryo developmental stage and O_2 dependent expression change in hepatocyte and cholangiocyte marker. Related to Figure 2 and 3; (A) Phase contrast imaging of E13.5 mouse liver tissue cultured with DMSO, 0.5 µM A83-01 or 10ng/mL TGFB3 for 72 hours (Scale bar, 1 mm). (B) Relative increased area of E13.5 mouse liver tissue cultured after 24 hours (mean±SD; n=6 independent experiments; *: P<0.05 vs. TGFB3). (C) Gene expression of E13.5 mouse liver tissue cultured after 72 hours (mean±SD; n=6 independent experiments; *: P<0.05 vs. TGFB3; §: P<0.05 vs. A83-01). (D) Change of oxygen exposure on human fetal development. Minimum and maximum oxygen concentrations are indicated blue belt. Mean oxygen concentrations are indicated black circle. Blood circulation is indicated gray line. Liver development events in each stage are indicated in the figure. This figure is created from various references (Tuuli et al., 2011; Morin 2017; Gregg et al., 1993; Gordillo et al., 2015). (E) Changes in O_2 concentration in culture medium with hiPSC-LBs (mean±SD; n=7~9 independent experiments; **P<0.01 vs. Mild-hypoxia). (F) Immunofluoresence staining for KRT19 (red), ALB (green) or SOX9 (green) in hiPSC-LB cultured for 5 days. Nuclei were stained using DAPI (blue). Scale bar, 100 µm (left side) and 20 µm (right side). (G) Analysis of hiPSC-LB in Figure S3F (mean±SD; n=6~8; 3 independent experiments; *: P<0.05 vs. Strong-hypoxia).

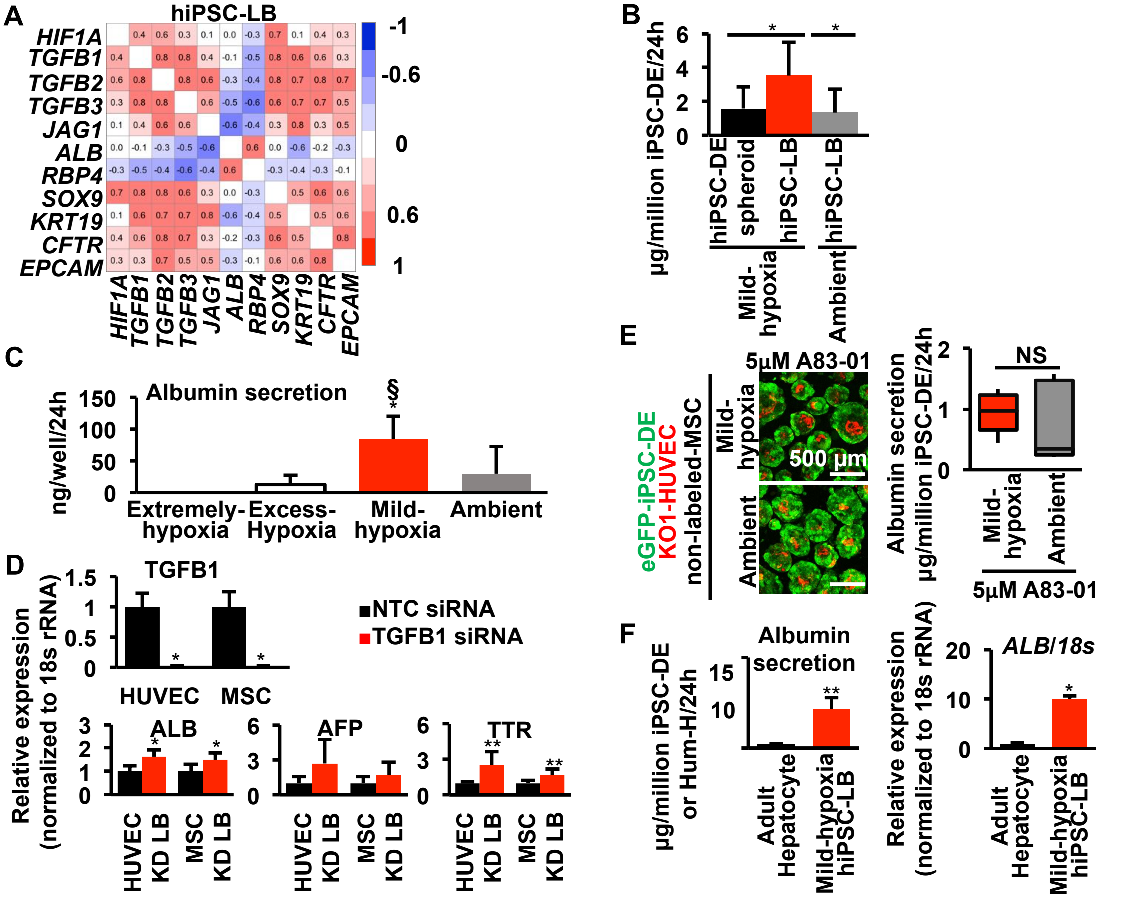

Figure S4. Correlation between O_2 and TGFB signal dependent expression change in hepatocyte and cholangiocyte markers, effect of stromal cells and O_2 in hiPSC-LB, and albumin level of adult hepatocyte. Related to Figure 3 and 4;
(A) Correlation analysis of hypoxia versus hepatocyte and cholangiocyte markers expressions in hiPSC-LB cultured for 10 days (n=36 independent experiments). (B) ELISA based albumin level of hiPSC-LB and hiPSC-DE spheroid cultured in Mild-hypoxia or Ambient for 15 days. The culture medium and growth factors are same as Figure 1E (mean±SD; n=7~22 independent experiments; *: P<0.05 vs. hiPSC-LB cultured in Mild-hypoxia). (C) Simultaneous comparison of albumin secretion of hiPSC-LBs cultured on day 10 in 4 conditions (mean±SD; n=8 or 9 independent experiments; * P<0.05 vs. Extremely-hypoxia; § P<0.05 vs. Excess-hypoxia). (D) Relative gene expression in TGFB1 knockdown (KD) study with siRNA treated-HUVEC/MSC and hiPSC-LB generated from TGFB1 KD HUVEC/MSC cultured on Excess-hypoxia (Top; mean±SD; n=6 independent experiments; *: P<0.05 vs. NTC siRNA, Bottom; mean±SD; n=8 independent experiments; *: P<0.05 and **: P<0.01 vs. hiPSC-LB generated from NTC siRNA treated HUVEC/MSC; NTC: Non-targeting control). (E) Inhibition of appropriate TGFB signal compensates O_2-dependent hepatic differentiation promotion in Mild-hypoxia (Left; Confocal images of hiPSC-LBs cultured in 5μM A83-01 for 15 days, green: eGFP-iPSC-DE cells (AAVS1::eGFP); red: KO1-HUVECs (MSCV-KO1); no label: MSCs; scale bar, 500 μm, Right; Boxplots of hiPSC-DE cell number normalized albumin level in hiPSC-LB on day15, The error bars represent the maximum and minimum values; n=12 independent experiments). The numbers of hiPSC-DE were calculated using the same rule as Figure 1E. (F) ELISA and gene expression based albumin level of adult hepatocyte and hiPSC-LB generated in Mild-hypoxia (Left; mean±SD; n=5or18 independent experiments; **: P<0.01 vs. hiPSC-LB generated in Mild-hypoxia; Right; n=5 or 6 independent experiments; *: P<0.05 vs. generated in Mild-hypoxia).

Supplemental Experimental Procedures

Animal

For mouse liver bud histology analysis, a female C57BL/6J mouse (SLC) on the tenth day of pregnancy was anesthetize with CO_2 and fetal mice before circulation (Swartley et al., 2016) were harvested rapidly for fixation. In the mouse liver tissue culture, female mice on the 13th day of pregnancy were anesthetized with CO_2 and fetal mice were harvested for liver dissection.

Measurement of pO_2, pCO_2, pH and glucose in hiPSC-LB culture medium

Partial pressure of oxygen (pO_2) in culture medium was measured by the Fibox3 single-channel fiber-optic oxygen meter (Presens) after 24 hours of last medium change. Partial pressure of carbon dioxide (pCO_2), pH and glucose consumption in culture medium were measured by Bioprofile 400 (kindly provided by Nova biomedical) after 24 hours of last medium change. We are not able to measure O_2 consumption by the cells due to the PDMS's higher O_2 diffusion coefficient. Therefore, we decided to measure dissolved O_2 in the medium instead of a direct measurement of O_2 consumption by the cells (Ramamoorthy et al., 2003, Kojima et al., 2015, Mattei et al., 2017 and Saito et al., 2006).

Immunostaining

hiPSC-LBs or mouse embryos were fixed in 4% paraformaldehyde (Wako) in phosphate buffered saline (PBS) for 15 minutes or overnight at 4 °C and infused in 30% sucrose (Wako) in PBS until sample sinks. Then the solution was replaced by 7.5% gelatin (Sigma) with 10% sucrose in PBS at 37°C for 15 minutes. The samples were embedded in new 7.5% gelatin with 10% sucrose in PBS and frozen with liquid N_2 after polymerization. Cryosections were placed on MAS-coated slides (Matsunami) for standard histological staining with hematoxylin/eosin (HE) or for immunostaining. For immunostaining, the samples were blocked by Protein Block Serum-Free (Dako), and were incubated with primary antibody at 4°C for overnight. Appropriate secondary antibodies conjugated to Alexa Fluor (Life Technologies) were incubated with the samples for 40 minutes at room temperature followed by DAPI (Sigma) nuclear staining. 0.05 % Tween20 (MP Biomedicals) in PBS was used for the sample washing and antibody dilution. The images were acquired using an Axio imager M1 microscope (Carl Zeiss). Image J software (NIH) was used to quantify the positive staining area.

Antibodies

Antibody	clone	Vendor	Catalog No.
Desmin		Lab Vision	RB-9014-P1
CD31	JC70A	Dako	M0823
Hif1a	H206	Santa Cruz Biotechnology	sc-10790
Hif2a		Novus Biologicals	NB100-122
Dlk	24-11	Medical & Biological Laboratories	D187-4
ALB		Sigma-Aldrich	A3293
AFP	C-19	Santa Cruz Biotechnology	sc-8108
KRT8/18		PROGEN	GP11
KRT19	RCK108	Dako	M0888
SOX9		EMD Millipore	AB5535

qRT-PCR and Microarray analysis

Total RNA was prepared using a PureLink RNA Mini Kit (Invitrogen). Then, qRT-PCR analyses and Microarray were conducted as described previously(Takebe et al., 2013). For qRT-PCR, total RNAs were reverse-transcribed with High-Capacity cDNA Reverse Transcription Kit (Applied Biosystems). 25ng of complementary DNAs (cDNAs) were used as qRT-PCR templates and mixed with gene specific primers and Taqman probe (Universal ProbeLibrary; Roche). Eukaryotic 18S rRNA Endogenous Control (Applied Biosystems) was used as reference and cycle reaction was performed using light cycler 480 systems (Roche). Gene expression levels were analyzed by ΔΔCp method. For microarray analysis, RNAs extracted from CD45-negative and Ter119-negative murine liver cells were hybridized on Whole Mouse Genome Agilent 4 X 44K v2 Oligonucleotide Microarray (Agilent Technologies) according to the manufacturer's instructions. Gene expression levels were analyzed using GeneSpring11.5.1.

qRT-PCR primers

Primer		sequence 5'>3'	Roche probe ID
ALB	Left	AATGTTGCCAAGCTGCTGA	27
	Right	CTTCCCTTCATCCCGAAGTT	
RBP4	Left	CCAGAAGCGCAGAAGATTG	17
	Right	TTTCTTTCTGATCTGCCATCG	
TGFB1	Left	CGACTACTACGCCAAGGAGGT	31
	Right	TGCTTGAACTTGTCATAGATTTCG	
TGFB2	Left	CAAAGGGTACAATGCCAACTT	67
	Right	GCAGATGCTTCTGGATTTATGG	
TGFB3	Left	AAGAAGCGGGCTTTGGAC	38
	Right	CGCACACAGCAGTTCTCC	
JAG1	Left	GGGCAACACCTTCAACCTC	28
	Right	GCCTCCACAAGCAACGTATAG	
INHBA	Left	CTCGGAGATCATCACGTTTG	72
	Right	CCTTGGAAATCTCGAAGTGC	
ANG1	Left	GACAGATGTTGAGACCCAGGTA	67
	Right	TCTCTAGCTTGTAGGTGGATAATGAA	
ANG2	Left	TGCAAATGTTCACAAATGCTAA	75
	Right	AAGTTGGAAGGACCACATGC	
ANGPTL4	Left	GTGGACCCTGAGGTCCTTC	18
	Right	CCACCTTGTGGAAGAGTTGC	
NODAL	Left	GGCGAGTGTCCTAATCCTGT	52
	Right	GCTGGTAACGTTTCAGCAGACT	

qRT-PCR primers

Primer		sequence 5'>3'	Roche probe ID
SOX9	Left	GTACCCGCACTTGCACAAC	61
	Right	TCTCGCTCTCGTTCAGAAGTC	
KRT19	Left	GCCACTACTACACGACCATCC	71
	Right	CAAACTTGGTTCGGAAGTCAT	
CFTR	Left	GAAGTAGTGATGGAGAATGTAACAGC	52
	Right	GCTTTCTCAAATAATTCCCCAAA	
HIF1A	Left	GAACCTGATGCTTTAACTTTGCT	28
	Right	TGCTGGTCATCAGTTTCTGTG	
EPCAM	Left	CCATGTGCTGGTGTGTGAA	3
	Right	TGTGTTTTAGTTCAATGATGATCCA	
mSox9	Left	GTACCCGCATCTGCACAAC	66
	Right	CTCCTCCACGAAGGGTCTCT	
VEGFA	Left	CGCAAGAAATCCCGGTATAA	1
	Right	AAATGCTTTCTCCGCTCTGA	

Albumin, AFP, Vitronectin, Ure, CYP3A4 and Genomic DNA measurement

To measure protein secretion level, HCM/EGM medium used for hiPSC-LB culture was collected after 24 hours from last medium change, and human Albumin Enzyme-Linked Immuno Sorbent Assay (ELISA) Quantitation Kit (Bethyl Laboratories), AFP (Human) ELISA Kit (Abnova) and Human Vitronectin ELISA Pair Set (Sino Biological) were used by according to the manufacturer's instructions. Absorbances of each sample were read by microplate reader, DTX880 (Beckman Coulter). On urea production assay, hiPSC-LBs were cultured in HCM/EGM medium for 16 days, and the medium was replaced to RPMI1640 including 80 ng/ml hepatocyte growth factor (HGF, Kringle Pharma), 2% B-27 Supplement, serum free, 1% MEM Non-Essential Amino Acids Solution and 100u/mL Penicillin-Streptomycin for next 4 days. Then the hiPSC-LBs were collected and cultured in the new medium with 2 mM NH_4Cl (Wako) on 96 wells plate. The medium was collected at 0 hrs and 6 hrs after hiPSC-LB re-seeding. Urea concentration in the medium was decided using QuantiChrom Urea Assay Kit (BioAssay Systems) used by according to the manufacturer's instructions. Absorbances of each sample were read by microplate reader, DTX880. To detect CYP3A4 activity, hiPSC-LBs were cultured in HCM/EGM medium for 15 days, and the medium was replaced to RPMI1640 including 80 ng/ml HGF, 2% B-27 Supplement, serum free, 1% MEM Non-Essential Amino Acids Solution and 100u/mL Penicillin-Streptomycin for next 2days.The medium was replaced to new medium with or without 25 μM Rifampicin (Wako) and cultured for 3 days. Then the hiPSC-LBs were collected and cultured in DMEM (high glucose) with L-glutamine and phenol red with 100u/mL Penicillin-Streptomycin, 0.1% CYP3A4-IPA (Promega), 1%

MEM Non-Essential Amino Acids Solution and 2% B-27 Supplement, serum free. The medium was collected after 6 hours and CYP3A4 activity was decided by CYP3A4 kit (Promega) used by according to the manufacturer's instructions. Luminescence of each sample were read by microplate reader, ARVO MX (Perkin Elmer). For genomic DNA quantification, collected hiPSC-LBs were incubated with 200mg/L Papain solution (Sigma-Aldrich) over night. Genomic DNAs in sample lysate were quantified by Quant-iT PicoGreen dsDNA Reagent (Invitogen) according to the manufacturer's instructions. Fluorescences of each sample were read by microplate reader, DTX880.

Sorting of hiPSC-LB

For performing fluorescence activated cell sorting (FACS), EGFP labeled iPSC-DE, KO1-labeled HUVEC and non-labeled MSC were co-cultured to generate hiPSC-LB. After 2 days, three wells of hiPSC-LBs (total 2.7E6 cells at the time of seeding) were pooled and wash with 1xPBS. The hiPSC-LBs were incubated with 2 mL of Accutase (Innovative Cell Technologies) about 1 hour at 37°C until cells were dissociated. Then the samples were centrifuged and re-suspended in 1mL of HCM/EGM. The dissociated cells were filtered with 35 µm nylon mesh (Falcon) and FACS-sorted by MoFlo Astrios (Beckman Coulter).

scRNA seq

The data were obtained from our previously published article (Camp et al., 2017) and can be downloaded from the NCBI Gene Expression Omnibus (GSE81252 and GSE96981). In brief, single cells dissociated from hiPSC-LB were captured on a microfluidic chip for mRNA-seq (Fluidigm) using a C1 system (Fluidigm), and SMARTer Ultra Low RNA kit for Illumina (Clontech) were used for cDNAs preparation on the chip. ERCC (External RNA Controls Consortium) RNA spike-in Mix (Invitrogen) was added to the lysis reaction and to process cellular mRNA at the same time. Sequencing libraries were constructed using a Nextera XT DNA Sample Preparation kit (Illumina) and each cell was sequenced using HiSeq 2500 (Illumina). Raw reads were mapped using TopHat and cDNAs were quantified as FPKM generated by Cufflinks. R script was used to perform PCA (FactoMineR package) and to construct violin plots.

TGFB1 Knock Down study

For TGFB1 knockdown study, 120 pmol of siRNA and 20 µL of Lipofectamine RNAiMAX Transfection Reagent (Thermo Fisher Scientific) were mixed in 2 mL of Opti-MEM I Reduced Serum Medium (Thermo Fisher Scientific) on 100 mm dishes and incubate for 20 minutes at room temperature. Then, 1.5E6 cells of HUVEC or 4.0E5 cells of MSC suspended in 8 mL of EGM (Lonza) or MSCGM (Lonza) without antibiotics were seeded before one day of co-culturing. These cells were co-cultured with hiPSC-DE for generating hiPSC-LB. After 4 days, these hiPSC-LBs were collected and quantified hepatic gene expression levels by qPCR analysis. The siRNAs used this study are siGENOME Non-Targeting siRNA Pool #2 (Dharmacon) or SMARTpool: siGENOME TGFB1 siRNA (Dharmacon).

Statistical Analysis

Mann-Whitney *U*-test was performed for 2 groups comparison. For multiple comparison, Mann-Whitney *U*-test with Bonferroni correction was performed after Kruskal Wallis

test. Pearson’s correlation was performed for verifying 2 groups correlation. Two-tailed p value of < 0.05 was considered as significant. Data are expressed as the mean±SD (Additional details were described in figure legends)

Supplemental References

Gregg, A.R., and Weiner, C.P. (1993) "Normal" umbilical arterial and venous acid-base and blood gas values. Clin Obstet Gynecol. 36, 24-32

Iwama, A., Oguro, H., Negishi, M., Kato, Y., Morita, Y., Tsukui, H., Ema, H., Kamijo, T., Katoh-Fukui, Y., Koseki, H. and van Lohuizen, M., (2004) Enhanced self-renewal of hematopoietic stem cells mediated by the polycomb gene product Bmi-1. Immunity, 21, 843-851.

Kojima, M., Takehara, H., Akagi, T., Shiono, H., and Ichiki, T. (2015) Flexible sheet-type sensor for noninvasive measurement of cellular oxygen metabolism on a culture dish. PloS one, 10, e0143774.

Mattei, G., Magliaro, C., Giusti, S., Ramachandran, S. D., Heinz, S., Braspenning, J., & Ahluwalia, A. (2017). On the adhesion-cohesion balance and oxygen consumption characteristics of liver organoids. PloS one 12, e0173206.

Morin, S.J. (2017) Oxygen tension in embryo culture: does a shift to 2% O2 in extended culture represent the most physiologic system? J Assist Reprod Genet. 34, 309-314

Ramamoorthy, R., Dutta, P. K., and Akbar, S. A. (2003) Oxygen sensors: materials, methods, designs and applications. Journal of materials science 38, 4271-4282.

Saito, T., Wu, C. C., Shiku, H., Yasukawa, T., Yokoo, M., Ito-Sasaki, T., Abe H., Hoshi H. and Matsue T. (2006) Oxygen consumption of cell suspension in a poly (dimethylsiloxane)(PDMS) microchannel estimated by scanning electrochemical microscopy. Analyst 131, 1006-1011

Serth, J., Kuczyk, M.A., Paeslack, U., Lichtinghagen, R. and Jonas, U. (2000) Quantitation of DNA extracted after micropreparation of cells from frozen and formalin-fixed tissue sections. The American journal of pathology, 156, 1189-1196.

Tuuli, M.G., Longtine, M.S., and Nelson, D.M. (2011) Review: Oxygen and trophoblast biology-a source of controversy. Placenta 32, S109-S118
