# Supplementary material for: Optimal Hypoxia Regulates Human iPSC-Derived Liver Bud Differentiation through Intercellular TGFB Signaling
Source: Stem Cell Reports. 2018 Jul 19;11(2):306–16. doi: 10.1016/j.stemcr.2018.06.015 (PMC6092760; doi:10.1016/j.stemcr.2018.06.015)
Supplement: Document S2. Article plus Supplemental Information [file mmc2.pdf]

Stem Cell Reports
Report

OPEN ACCESS

Optimal Hypoxia Regulates Human iPSC-Derived Liver Bud Differentiation through Intercellular TGFB Signaling

Hiroaki Ayabe,[1] Takahisa Anada,[2] Takuo Kamoya,[2] Tomoya Sato,[2] Masaki Kimura,[4] Emi Yoshizawa,[1] Shunyuu Kikuchi,[1] Yasuharu Ueno,[1] Keisuke Sekine,[1] J. Gray Camp,[3] Barbara Treutlein,[3] Autumn Ferguson,[4] Osamu Suzuki,[2] Takanori Takebe,[1,4,5,6,*] and Hideki Taniguchi[1,*]

[1]Department of Regenerative Medicine, Yokohama City University Graduate School of Medicine, Kanazawa-ku 3-9, Yokohama, Kanagawa 236-0004, Japan
[2]Division of Craniofacial Function Engineering, Tohoku University Graduate School of Dentistry, 4-1 Seiryo-machi, Aoba-ku, Sendai 980-8575, Japan
[3]Max Planck Institute for Evolutionary Anthropology, Leipzig 04103, Germany
[4]Division of Gastroenterology, Hepatology & Nutrition, Developmental Biology, Center for Stem Cell and Organoid Medicine (CuSTOM), Cincinnati Children's Hospital Medical Center, 3333 Burnet Avenue, Cincinnati, OH 45229-3039, USA
[5]Department of Pediatrics, University of Cincinnati College of Medicine, 3333 Burnet Avenue, Cincinnati, OH 45229-3039, USA
[6]Institute of Research, Tokyo Medical and Dental University, 1-5-45 Yushima, Bunkyo-ku, Tokyo 113-8510, Japan
*Correspondence: takanori.takebe@cchmc.org (T.T.), rtanigu@yokohama-cu.ac.jp (H.T.)
https://doi.org/10.1016/j.stemcr.2018.06.015

SUMMARY

Timely controlled oxygen (O_2) delivery is crucial for the developing liver. However, the influence of O_2 on intercellular communication during hepatogenesis is unclear. Using a human induced pluripotent stem cell-derived liver bud (hiPSC-LB) model, we found hypoxia induced with an O_2-permeable plate promoted hepatic differentiation accompanied by *TGFB1* and *TGFB3* suppression. Conversely, extensive hypoxia generated with an O_2-non-permeable plate elevated *TGFBs* and cholangiocyte marker expression. Single-cell RNA sequencing revealed that *TGFB1* and *TGFB3* are primarily expressed in the human liver mesenchyme and endothelium similar to in the hiPSC-LBs. Stromal cell-specific RNA interferences indicated the importance of TGFB signaling for hepatocytic differentiation in hiPSC-LB. Consistently, during mouse liver development, the *Hif1a*-mediated developmental hypoxic response is positively correlated with *TGFB1* expression. These data provide insights into the mechanism that hypoxia-stimulated signals in mesenchyme and endothelium, likely through TGFB1, promote hepatoblast differentiation prior to fetal circulation establishment.

INTRODUCTION

Organoid technology using human pluripotent stem cells or isolated organ progenitors is rapidly evolving to model the elaborate spatiotemporal processes of development and regeneration. Progenitors are directed to self-organize into an organoid comprising multiple cell types, including epithelial and mesenchymal cell lineages (Lancaster and Knoblich, 2014). Because human organoids recapitulate certain aspects of native organ architecture that are highly inaccessible, organoid technology has recently emerged as an essential tool for both human biology and pathology research (Kretzschmar and Clevers, 2016). For example, a human induced pluripotent stem cell-derived liver bud (hiPSC-LB) can be generated by co-culture with hiPSC-derived liver progenitor cells, human umbilical vein endothelial cells (HUVECs), and human mesenchymal stem cells (MSCs) (Takebe et al., 2013 and 2017). The complex self-organized hiPSC-LB is an organ-like 3D tissue with cell-cell interactions that recapitulate early LB structures and transcriptomic signatures (Camp et al., 2017). However, *in vitro* organoids generally remain developmentally immature without transplantation into animals (Dye et al., 2016; Xinaris et al., 2012; Watson et al., 2014), highlighting the need for filling the maturation gaps between *in vitro* and *in vivo* systems.

Developmental hypoxia is considered a critical phenomenon, especially in early phases of organogenesis prior to blood perfusion. For example, the mammalian embryo develops in a low-O_2 environment, and in this context, hypoxia-inducible factor (HIF) has additional responsibilities in multiple processes (Dunwoodie, 2009). In vertebrate models, the developing LB expresses HIF2A prior to the initiation of fetal circulation (Lin et al., 2014), indicating that early hepatoblast expansion and delamination require transient exposure to hypoxic conditions. Similarly, in the developing kidney, both HIFA isoforms are activated in a cell-specific and temporally controlled manner, indicating a regulatory role for O_2 tension in nephrogenesis (Bernhardt et al., 2006). These reports suggest that physiological hypoxia potentially impacts early organ development.

The aim of this study was to evaluate the role of O_2 conditions in directing liver organoids and early liver development. Specifically, to clarify the precise hypoxic role in liver development, we characterized the influence of variable O_2 tensions with O_2-permeable culture plates using hiPSC-LBs as a fetal liver model.

 © 2018 The Authors.
This is an open access article under the CC BY license (http://creativecommons.org/licenses/by/4.0/).

RESULTS

Hypoxic Conditions Generated with an O_2-Permeable Plate Promoted Hepatocyte Differentiation in Liver Buds

As the use of O_2-impermeable culture plates that applied the strong hypoxic environment, a polydimethylsiloxane (PDMS) plate was used due to its stability and tunability of O_2 conditions (Figures 1A, 1B, and S1A). All of the hypoxic culture conditions assessed in this study are summarized in Figure S1A.The most important feature of this plate is that it provides a direct O_2 supply to the cells because the plate is made of PDMS that is only permeable to gas (Anada et al., 2012), promoting the differentiation and survival of 3-D cultured MSCs (Anada et al., 2016; Kamoya et al., 2016). Conversely, a lower O_2 level incurred using polystyrene plates with low O_2 permeability, because PDMS's O_2 permeability limits the ability to control low O_2 tension (Byrne et al., 2014). Excess-hypoxia condition enables a comparison between test sets. Mild-hypoxia (10% O_2, O_2-permeable plate) and Ambient (20% O_2, O_2-permeable plate) condition equilibrate culture medium with external O_2 tension, with minimum values of 13% and 19%, respectively (Figure 1B). However, dissolved O_2 modulated with Excess-hypoxia condition (20% O_2, O_2-non-permeable plate) had the lowest (10%) O_2 levels under all conditions during the first 3 to 4 days and then the O_2 tension gradually increased. Although the CO_2 level in the medium of Ambient condition was slightly low, the pH value in all groups was stable at approximately pH 7 (Figure S1B). Thus, the PDMS plate is adequate for evaluation of higher O_2 conditions.

To induce hiPSC-LB self-organization, hiPSC-derived definitive endoderm cells (hiPSC-DE) were mixed with HUVECs and MSCs (Figure 1A). Then, the cell mixture was seeded onto micro-patterned plates and cultured under various O_2 conditions. After 15 days of culture, HUVECs in hiPSC-LBs cultured on Mild-hypoxia and Ambient condition formed a network structure, and MSCs were observed to be located adjacent to HUVECs (Figure 1C). Moreover, hiPSC-LBs cultured on Mild-hypoxia condition showed the highest median diameter (Figure 1D). In addition, these hiPSC-LBs exhibited the highest average LB number per well and the highest diameter ratio (over 150 μm) (Figures S1C and S1D).

In order to extrapolate the ratio of hiPSC-DE and stromal cells (HUVECs and MSCs) in hiPSC-LB cultured for 15 days, we counted CK8/18 stained cells divided by DAPI-stained nuclei to estimate hiPSC-DE cell percentage (Figure S1E). Although the initial ratio of hiPSC-DE and stromal cells were 1 and 0.9 in hiPSC-LB, the eventual ratio of hiPSC-DE and stromal cell in hiPSC-LBs was 0.91 versus 0.09 in Excess-hypoxia, 0.66 versus 0.34 in Mild-hypoxia and 0.83 versus 0.17 in Ambient on day 15 in immunohistochemistry of respective lineage makers (Figure S1E). Combined with total genomic DNA amount (Figure S2E), we estimate hepatic cell numbers in a well that were 1.5×10^5, 1.8×10^5, and 1.5×10^5 in Excess-hypoxia, Mild-hypoxia, and Ambient, which are approximately 30% of seeding time and calculated based on a published literature, which estimates from the genomic DNA amount in a cell at 6.57 pg/cell (Figure S2F). Given that almost all hiPSC-LBs were collected (we established collection rate of >98% as criterion), the reduction of hiPSC-DE suggests presence of cell death in hiPSC-LB associated with long-term culture. Additionally, the stromal cell numbers in a well were 0.14×10^5, 0.93×10^5, and 0.32×10^5 in Excess-hypoxia, Mild-hypoxia, and Ambient, although the number was 4.3×10^5 at the seeding time. Therefore, the hypoxic culture in Mild-hypoxia group most efficiently preserved stromal lineage in hiPSC-LB.

Regarding average albumin secretion value normalized by the number of hiPSC-DE cells in a well, the Mild-hypoxia group (8.6 μg/million hiPSC-DE/24 hr) was approximately six times higher than the Excess-hypoxia group (1.4 μg/million hiPSC-DE/24 hr) and approximately two times higher than the Ambient group (4.1 μg/million hiPSC-DE/24 hr) on day 15 (Figure 1E). The secretion of vitronectin (a cell adhesion protein) in the Mild-hypoxia group (6.9 μg/million hiPSC-DE/24 hr) was also significantly higher than the Ambient group (1.7 μg/million hiPSC-DE/24 hr), although the secretion of alpha-fetoprotein (AFP; a fetal liver marker) was not significant. LBs cultured on Mild-hypoxia condition also demonstrated an increase in the basal level of cytochrome P450 family 3 subfamily A member 4 (CYP3A4) activity and urea production (Figures S2A and S2B). Additionally, immunofluorescence indicated that all the liver markers examined were expressed in all groups (Figure S2C). *ALB* and *RBP4* gene expression levels normalized to 18s rRNA expression of hiPSC-DE in hiPSC-LB were higher in the LBs cultured on Mild-hypoxia and Ambient condition than those in the LBs grown on Excess-hypoxia condition (Figure S2D). These data suggest that Mild-hypoxia promotes human hepatoblast differentiation in hiPSC-LBs rather than just a proliferation.

TGFB Signals from the Mesenchyme and Endothelium Are Candidate Regulators of O_2-Dependent Hepatocyte Differentiation in LBs

In embryonic day 13.5 (E13.5) mice, the hepatoblasts starts to differentiate into either hepatocytes or cholangiocytes (Zorn, 2008), and TGFBs and JAGGED1 (JAG1) direct the fate of these cells by promoting hepatoblast differentiation into cholangiocytes (Zorn, 2008; Gordillo et al., 2015). TGFB inhibits the proliferation of both hepatocytes and

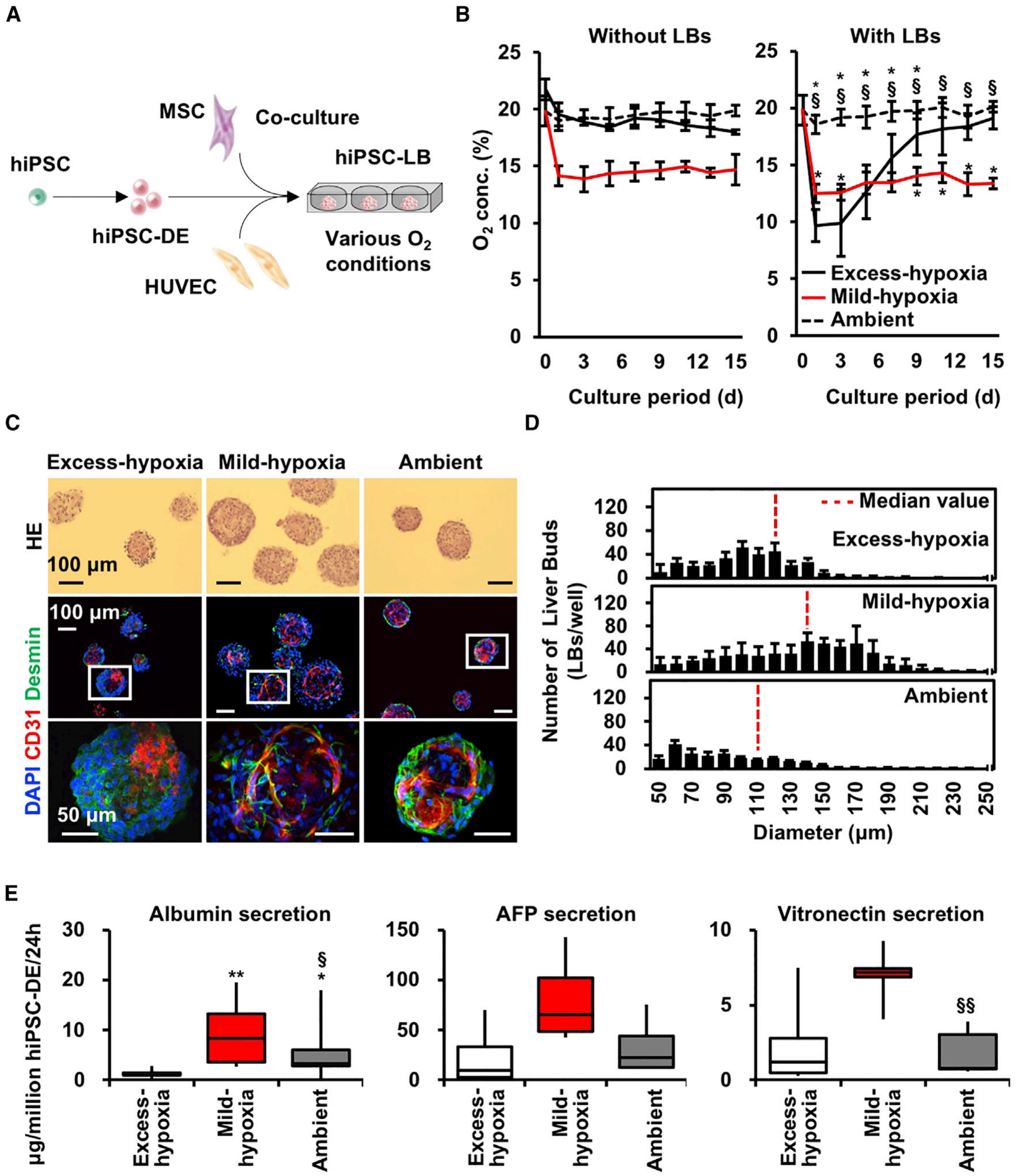

Figure 1. Hypoxic Conditions Generated with an O_2-Permeable Plate Promoted Hepatocyte Differentiation in Liver Buds

(A) Schematic view of the present study.

(B) Changes in O_2 tension in culture medium without (left) or with (right) hiPSC-LBs for 15 days (mean ± SD; n = 8 or 9 independent experiments; $^{*}p < 0.05$ versus Excess-hypoxia; $^{\S}p < 0.05$ versus Mild-hypoxia).

(C) H&E and immunofluorescence staining of hiPSC-LBs cultured for 15 days (CD31: red; Desmin: green; nuclei: blue [DAPI]). Scale bar, 100 μm (upper and middle side) or 50 μm (lower side).

(D) Diameter distribution of hiPSC-LBs cultured for 15 days (mean ± SD; n = 9–11 independent experiments).

(E) Boxplots of hiPSC-DE cell number normalized protein level in hiPSC-LB on day15. The error bars represent the maximum and minimum values; n = 8–18 independent experiments; $^{*}p < 0.05$ and $^{**}p < 0.01$ versus Excess-hypoxia; $^{\S}p < 0.05$ and $^{\S\S}p < 0.01$ versus Mild-hypoxia.

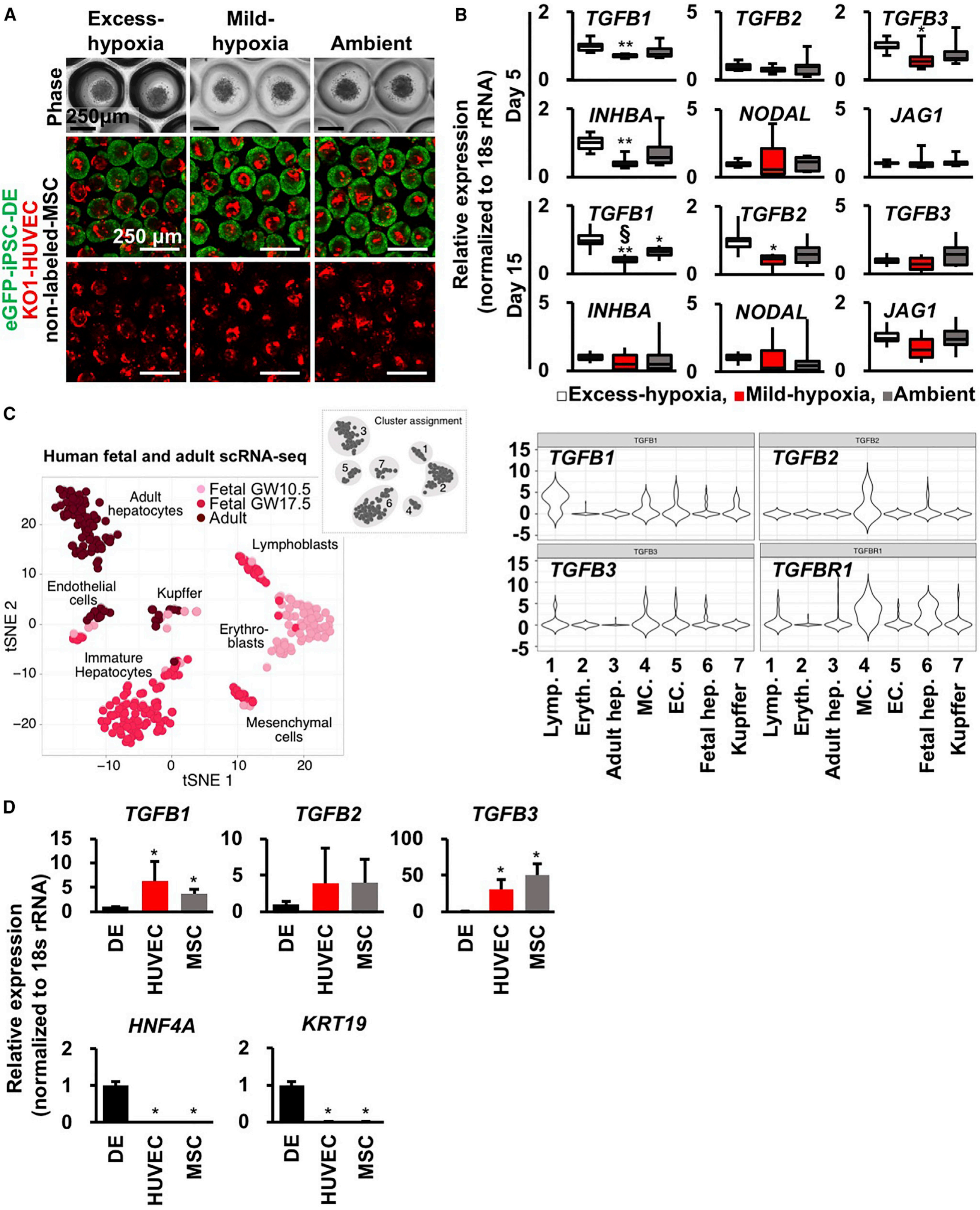

(legend on next page)

endothelial cells (ECs) (Nguyen et al., 2007; Takehara et al., 1987) and hepatocyte differentiation (Spagnoli et al., 2000). Additionally, TGFB induces apoptosis in both hepatocytes and ECs (Black et al., 2007; Ferrari et al., 2012). Consistently, our fetal mouse liver organ culture indicated that TGFB3 inhibits liver outgrowth, and TGFB3 signaling inhibition suppresses *Sox9* (cholangiocyte marker) gene expression (Figures S3A–S3C). Given that early hepatic organogenesis is accompanied by TGFB modulation across multiple cell types, we hypothesized that Mild-hypoxia condition-induced hepatocyte differentiation occurred in hiPSC-LBs by means of TGFB reduction.

To analyze the role of variable O_2 condition on TGFBs and JAG1, we quantified gene expression in the hiPSC-LBs on days 5 and 15 (Figures 2A and 2B). The TGFB1, TGFB3, and inhibin beta A subunit (INHBA) gene expression levels in hiPSC-LBs grown on Mild-hypoxia was significantly lower than those in hiPSC-LBs cultured on Excess-hypoxia (*TGFB1*, 0.7; *TGFB3*, 0.6; *INHBA*, 0.4 versus Excess-hypoxia; mean of gene expression) on day 5. Moreover, the TGFB1 gene expression levels in hiPSC-LB cultured on Mild-hypoxia was significantly lower than the Ambient group on day 15 (0.4; versus Ambient; mean of gene expression). Additionally, the O_2 concentration in Mild-hypoxia group is also significantly lower than Ambient group on day 15. Therefore, the Mild-hypoxia condition is the most efficient in reducing TGFB expression and provides a favorable environment for hepatocytic differentiation.

To determine which cells produce TGFBs in the human liver, we reanalyzed the gene expression of human liver using single-cell RNA sequencing (Camp et al., 2017) (Figure 2C). The *TGFB2* gene was clearly expressed in mesenchymal cells (MCs) in the liver, and both *TGFB1* and *TGFB3* were expressed in ECs and MCs. Moreover, TGFB receptor 1 (TGFBR1) was clearly expressed in fetal hepatocytes and MCs. We then performed fluorescence-activated cell sorting (FACS) using dissociated hiPSC-LBs to investigate the cellular source for TGFB ligands (Figure 2D). Interestingly, *TGFB1* and *TGFB3* gene expression levels were significantly higher in isolated HUVECs and MSCs than those in hiPSC-DE cells. In addition, hepatocyte nuclear factor 4 alpha (*HNF4A*; hepatocyte marker) and cytokeratin 19 (*KRT19*; cholangiocyte marker) expression levels were higher in hiPSC-DE cells than those in other cells. These results indicate that HUVECs and MSCs produce major TGFB1 and 3 in hiPSC-LBs. This suggests that the major source of TGFB supply in hiPSC-LBs seems consistent with that in human fetal liver cells. Given that the signal from the portal MCs and/or portal vein ECs is a candidate promoter of hepatoblast maturation toward cholangiocytes (Clotman et al., 2005), balanced TGFB1 and 3 signals from MCs and ECs are likely regulators of O_2-dependent hepatocyte differentiation in the LB.

Hif1a Is Positively Correlated with *Tgfbs* and Biliary Markers and Negatively Correlated with Hepatocyte Markers in Mouse Liver Development

To investigate whether O_2-dependent LB development is present *in vivo*, we compared mouse fetal liver microarray analyses on multiple embryonic days (Figure 3A). The mouse LB is established before E10 (Zorn, 2008; Gordillo et al., 2015), and blood circulation is initiated and sophisticated after LB generation (Swartley et al., 2016). Therefore, *Hif1a* expression is hypothetically influenced by O_2 tension change through fetal circulation development. During early hepatogenesis, along with the gradual reduction of *Tgfb* gene expression, cholangiocyte markers of SRY ([sex determining region Y]-box 9 [*Sox9*]) and cystic fibrosis transmembrane conductance regulator (*Cftr*) decreased, whereas hepatocyte markers, such as *Alb* and *Rbp4*, increased over time. Immunofluorescence indicated the presence of HIF1A and HIF2A expression in E10.5 fetal livers (Figure 3B). These data indicate that mouse LB development is initiated under potent hypoxic conditions and hepatic gene expression is increased after liver circulation develops. Correlation analysis indicated that *Hif1a*; *Tgfb*1, 2, and 3; and biliary markers were positively correlated, and *Hif1a* and hepatocyte markers were negatively correlated (Figure 3C). Thus, hepatic and

Figure 2. TGFB Signals from the Mesenchyme and Endothelium Are Candidate Regulators of O_2-Dependent Hepatocyte Differentiation in Liver Buds

(A) Phase-contrast and confocal images of hiPSC-LBs cultured for 1 (phase) or 5 (confocal) days (green: eGFP-iPSC-DE cells [AAVS1:EGFP]; red: KO1-HUVECs [MSCV-KO1]; no label: MSCs; scale bar, 250 μm).

(B) Boxplots of TGFB family gene expression in hiPSC-LBs cultured for 5 and 15 days. The error bars represent the maximum and minimum values; n = 9 (day 5) and 10 (day 15) independent experiments; *p < 0.05 and **p < 0.01 versus Excess-hypoxia; §p < 0.05 versus Ambient.

(C) Left: tSNE of single-cell transcriptomes from human fetal (two donors, gestation weeks 10.5 and 17.5, 238 cells) and adult (three donors, age 21–65, 256 cells) liver samples (modified from Camp et al., 2017). Right: violin plots of single-cell transcriptomes, showing the distribution in the human liver.

(D) Gene expression of each cell lineage in hiPSC-LB. hiPSC-LBs were cultured on Ambient group for 2 days. Then, dissociated eGFP-DE cells, KO-HUVECs, and unlabeled-MSCs from hiPSC-LBs were separated by FACS analysis using fluorescence labels. Gene expression of these cells was analyzed (mean ± SD; n = 8 independent experiments; *p < 0.05 versus hiPSC-DE cells).

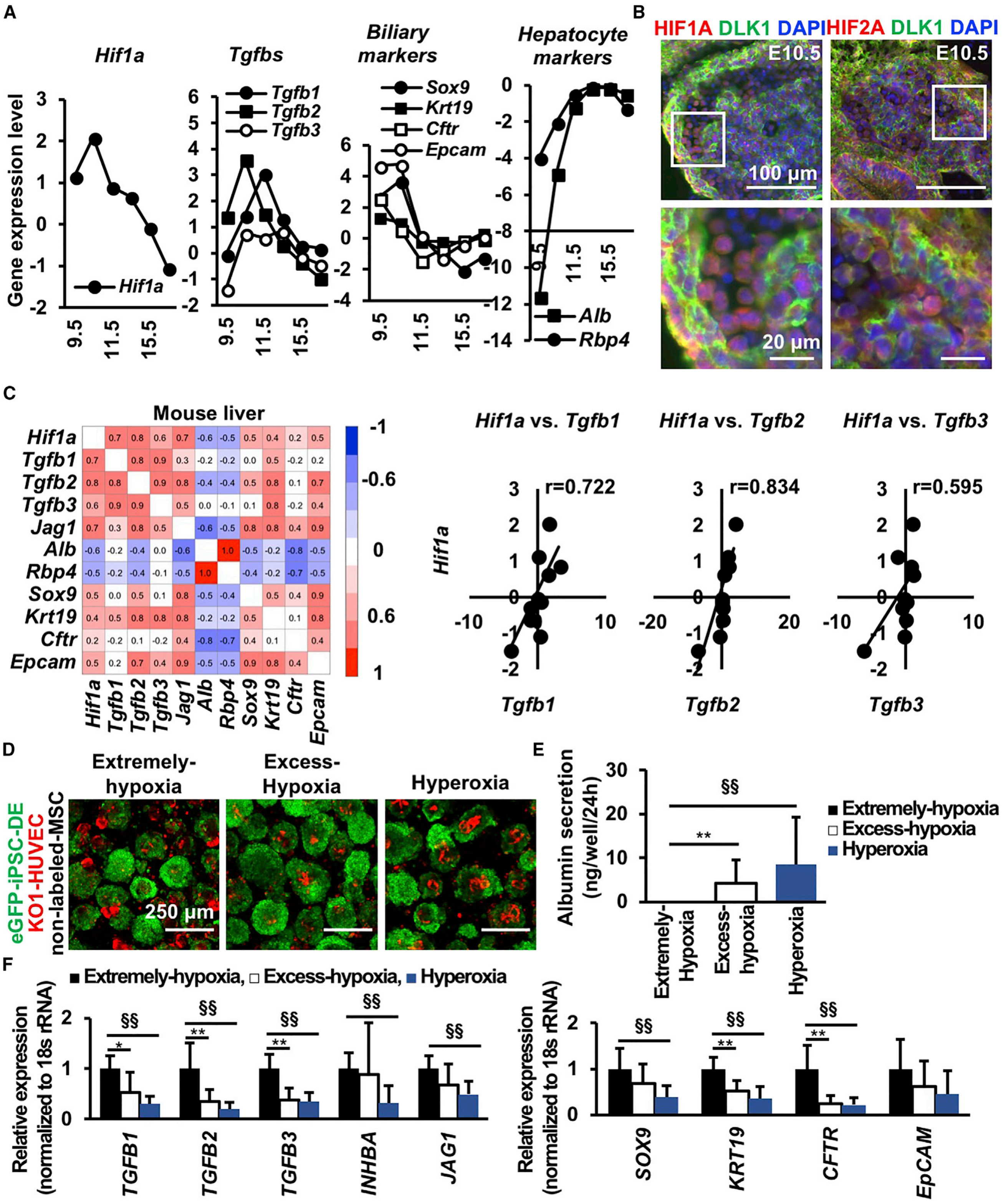

Figure 3. *Hif1a* Is Positively Correlated with *Tgfbs* and Biliary Markers and Negatively Correlated with Hepatocyte Markers in Mouse Liver Development

(A) Microarray gene expression analysis of mouse fetal liver from E9.5 to 17.5.

(legend continued on next page)

biliary gene expressions were influenced by hypoxic signatures *in vivo*.

Considering the potential O_2-dependent TGFB modulation in the *in vitro* LB model, the effect of stronger hypoxic conditions in LB differentiation was investigated (Figures 3D and S3E). Compared with the hiPSC-LBs cultured under Excess-hypoxia and/or hyperoxia conditions, those cultured under Extreme-hypoxia conditions exhibited downregulated albumin secretion (Figure 3E) and upregulated *TGFB* family genes and cholangiocyte marker gene expression (Figure 3F). Immunofluorescence also indicated that, compared with Strong-hypoxia and Mild-hypoxia condition, hypoxic conditions increase cholangiocyte marker expression and decrease hepatocyte marker expression (Figures S3F and S3G). Additionally, correlation analysis of *HIF1A*, *TGFB*, biliary marker, and hepatocyte marker gene expression in hiPSC-LBs indicated a pattern similar to that in the mouse liver (Figures 3C and S4A). These data suggest the appropriate hypoxic condition permits hepatoblast lineage commitment through the modulation of TGFB signaling.

To investigate whether MSCs and HUVECs in hiPSC-LB is necessary for influencing hepatoblast differentiation through TGFB and O_2 tension, we investigated hepatic function of 3D cultured hiPSC-DE cells. Albumin secretion is two times lower by hiPSC-DE spheroids than hiPSC-LB in Mild-hypoxia condition (Figure S4B), suggesting the presence of stromal cells significantly augments hepatic differentiation in hypoxic condition. Hepatic differentiation again measured by albumin levels in Extreme-hypoxia and Excess-hypoxia group are significantly lower than in the Mild-hypoxia group (Figure S4C). In agreement with a previous study (Hung et al., 2013), the expression level of TGFB1 at day 5 in Excess-hypoxia is significantly higher than Mild-hypoxia (Figure 2B). A previous literature reported that the half-life of TGFB1 mRNA is 15 hr (Bascom et al., 1989). Therefore, TGFB1 gene expression level at day 5 is likely affected by the day 3 to 4 condition.

To highlight the cell-type-specific mechanism for promoting hepatic differentiation, we interfered TGFB1 gene in stromal lineages (HUVEC or MSC) of hiPSC-LB in Excess-hypoxia condition (Figure S4D). Gene expression of *ALB* and *TTR* in TGFB1 knocked down hiPSC-LB is approximately 1.5 to 2.5 times higher than control in Excess-hypoxia condition at day 4. On day 15, TGFB1 gene expression and O_2 concentration in the Ambient group is higher than in the Mild-hypoxia group, and albumin secretion level in the Ambient group is lower than in the Mild-hypoxia group (Figures 2B, 1B, and 1E). Thus, upregulated TGFB1 expression level relative to the Mild-hypoxia condition likely impairs hepatic differentiation. These data suggest that optimal hypoxia regulates hiPSC-LB differentiation through intercellular TGFB signaling.

TGFB Signal Inhibition Promotes Hepatocyte Differentiation in LBs

To investigate the effects of TGFB on hiPSC-LBs, we blocked TGFB signaling using the TGFB inhibitor A83-01 in the Excess-hypoxia group (Figures 4A and 4B). The hiPSC-LBs were collected on day 15 and Kusabira-Orange fluorescent protein (KO1) fluorescence intensity was measured to quantify the abundance of KO1-labeled HUVECs. As expected, blocking TGFB signaling gradually increased the abundance of HUVECs at an A83-01 concentration of up to 0.5 μM, and the HUVEC abundance was three times higher than that in the control. The rate of hiPSC-LBs, which exhibit high KO1 fluorescence intensity, was 5% in 0 μM A83-01 and 55% in 0.5 μM A83-01. The expression level of angiogenesis-related cytokines, such as vascular endothelial growth factor A (*VEGFA*), angiopoietin2 (*ANG2*), and angiopoietin-like 4 (*ANGPTL4*) (Figure 4C) increased under hypoxic conditions and was positively correlated with that of *TGFB*s. TGFBs induce EC apoptosis mediated by VEGF signaling (Ferrari et al., 2012); ANG2 antagonizes the vascular receptor tyrosine kinase (Augustin et al., 2009), and ANGPTL4 inhibits EC adhesion, migration, and sprouting (Cazes et al., 2006). These data suggest that O_2 tension and TGFBs influence angiogenesis in hiPSC-LBs. In addition, gene expression and protein secretion of hepatocyte makers in hiPSC-LBs in the Excess-hypoxia group gradually increased in a dose-dependent manner (Figures 4D and 4E). On day 15, *ALB* gene expression was the highest in 0.5 μM A83-01-treated LBs and *RBP4* expression was highest in 5 μM A83-01-treated LBs. Albumin secretion was also increased with concentrations up to 0.5 μM A83-01, and the value was 1.5 μg/well/24 hr,

(B) Immunofluorescence staining for HIF1A (red), HIF2A (red), DLK1 (green), and nuclei (blue, DAPI) in E10.5 mouse liver. Scale bar, 100 μm (upper) or 20 μm (lower).

(C) Correlation analysis of hypoxia- (*Hif1a*), hepatocyte- (*Alb* and *Rbp4*), and cholangiocyte- (others) associated markers in mouse livers from E9.5 to 8-week-old mice.

(D) hiPSC-LBs cultured for 10 days (green: eGFP-iPSC-DE cells [AAVS1:EGFP]; red: KO1-HUVECs [MSCV-KO1]; no label: MSCs; scale bar, 250 μm).

(E) ELISA on protein secretion in hiPSC-LBs cultured for 10 days (mean ± SD; n = 15 independent experiments; $^{**}p < 0.01$ versus Excess-hypoxia, $^{§§}p < 0.01$ versus Extreme-hypoxia).

(F) Gene expression in hiPSC-LBs cultured for 10 days (mean ± SD; n = 12 independent experiments; $^{*}p < 0.05$ and $^{**}p < 0.01$ versus Excess-hypoxia, $^{§§}p < 0.01$ versus Extreme-hypoxia).

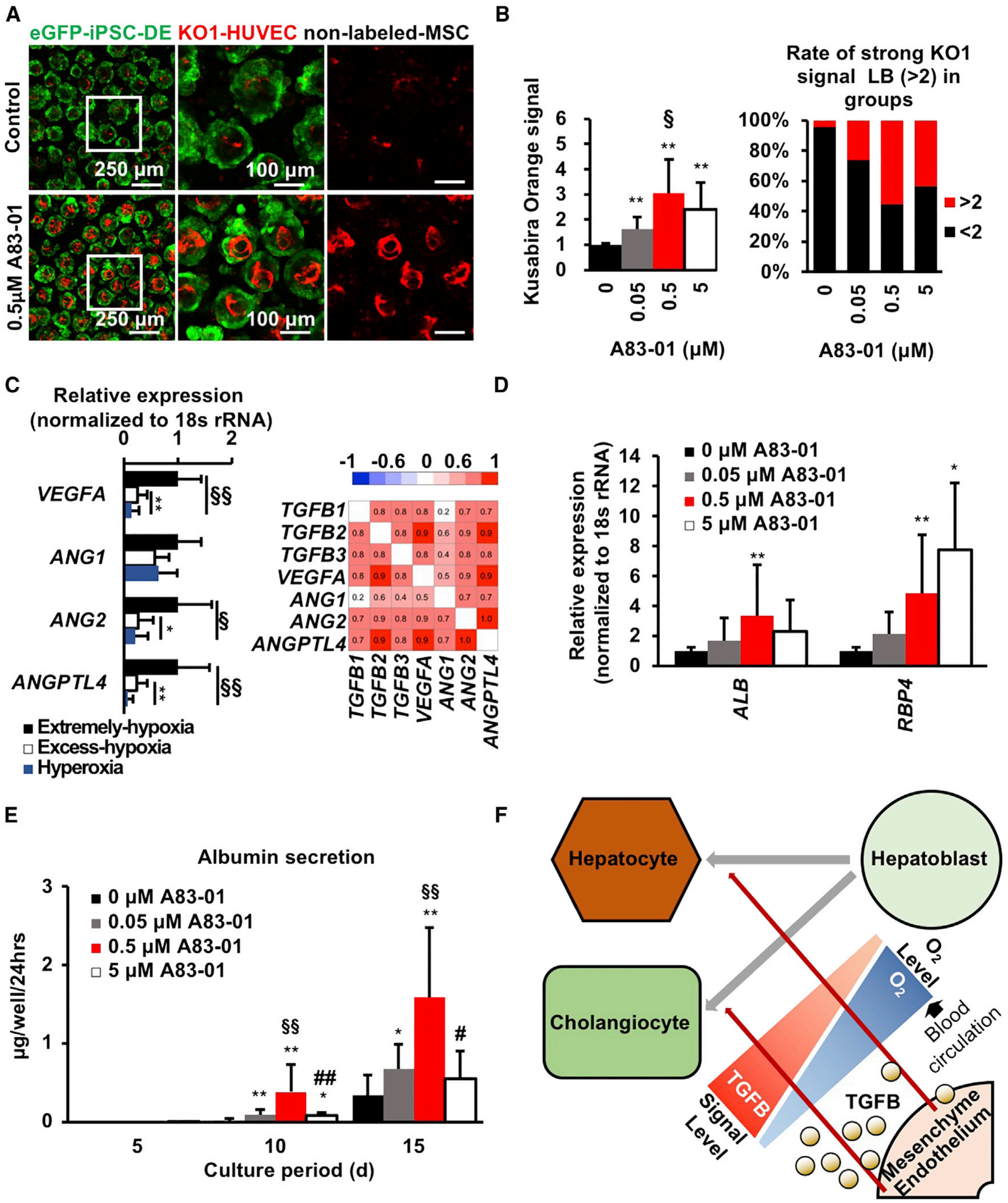

Figure 4. TGFB Signal Inhibition Promotes Hepatocyte Differentiation in Liver Buds

(A) Confocal imaging of hiPSC-LBs cultured with various concentrations of A83-01 for 15 days in Excess-hypoxia group (green: eGFP-iPSC-DE cells [AAVS1:EGFP]; red: KO1-HUVECs [MSCV-KO1]; no label: MSCs; scale bar from left to right, 250, 100, and 100 μm).

(B) Image analysis of HUVEC abundance in hiPSC-LBs cultured with various A83-01 concentrations for 15 days in Excess-hypoxia group. Fluorescence intensity of KO1 protein expression in HUVECs was evaluated as HUVEC abundance in hiPSC-LBs (left: mean ± SD; n = 9–17 independent experiments; **p < 0.01 versus 0 μM; §p < 0.05 versus 0.05 μM; right: a total of 1449–2985 LBs were measured).

(C) Gene expression and correlation of hiPSC-LBs cultured for 10 days (mean ± SD; n = 12 independent experiments; *p < 0.05 and **p < 0.01 versus Excess-hypoxia, §p < 0.05 and §§p < 0.01 versus Extreme-hypoxia).

(D) Gene expression in hiPSC-LBs cultured with various A83-01 concentrations for 15 days in Excess-hypoxia group (mean ± SD; n = 6–14 independent experiments; *p < 0.05 and **p < 0.01 versus 0 μM).

(legend continued on next page)

which was five times higher than that in the control. However, 5 μM A83-01 induced a significant reduction in albumin secretion and HUVEC abundance as well as a reduction in *ALB* gene expression. Finally, we compared the albumin levels in hiPSC-LBs with those in frozen adult hepatocytes (Figure S4F). Albumin secretion and gene expression levels in hiPSC-LBs on the Mild-hypoxia condition was 10-fold higher than those of frozen adult hepatocytes. These data suggest that appropriate TGFB signals derived from HUVECs and MSCs promote hepatocyte differentiation in LBs. These data support a potential mechanism of O_2-dependent differentiation in human LBs through TGFB signaling (Figure 4F).

DISCUSSION

All three TGFB isoforms (TGFB1, 2, and 3) are known to induce biliary marker expression and repress hepatocyte marker expression in mouse hepatoblasts (Antoniou et al., 2009). *Tgfb1* mRNA was expressed widely in the fetal mouse liver, whereas *Tgfb2* and *Tgfb3* mRNA was found predominantly in the periportal region (Antoniou et al., 2009). In the present study, *Tgfb1* expression in fetal mouse liver decreased in a time-dependent manner from the initiation of blood circulation with a positive correlation to the hypoxic response. Therefore, this *Tgfb1* reduction stimulates differentiation of hepatoblasts into hepatocytes rather than cholangiocytes coincided by fetal circulatory system establishment.

Additionally, excess TGFB inhibition impaired hiPSC-LB differentiation, suggesting optimal TGFB expression in HUVECs and MSCs is important for hepatocyte differentiation. Although the reason is uncertain, TGFB also has a positive effect on fetal hepatocyte development. A previous study showed that TGFB upregulates fibronectin expression in mouse embryos (Sugiyama et al., 2013), and fibronectin upregulates hepatic gene expression in rat fetal hepatocytes (Sanchez et al., 2000).

During liver regeneration, the TGFB family cytokine activin A works as a negative regulator of hepatocyte proliferation and serves as a candidate terminator of liver regeneration (Bohm et al., 2010). We previously reported that iPSC-DE cells co-cultured with HUVECs or MSCs using a 2D culture system exhibit decreased albumin production and hepatic maturation (Asai et al., 2017). Hypoxic factors, including INHBA (an activin subunit), are candidate factors for explaining this discrepancy. Mild-hypoxia (PDMS plate in 10% O_2) condition reduced the gene expression of *INHBA* and other *TGFB* family members. Moreover, the activin A signal is a target of A83-01. Although further mechanistic investigation of other possible factors linking hypoxia with hepatic differentiation in developing liver will be needed, optimized O_2 conditions together with multicellular co-culture will be a potential strategy to promote maturation into the hepatocytic lineage.

Collectively, timely control of hypoxia induction to hiPSC-LB or mouse LB promoted the stable hepatocytic differentiation. However, it remains unclear how the spatial gradient of developmental hypoxia contributes early differentiation in LB. Toward this goal, combinatorial use of a tissue engineering approach will be promising for enabling *in vitro* optimized O_2 delivery such as through a perfusion device. Future efforts to optimize spatiotemporal O_2 control will produce functional and reproducible liver organoids, which will be advantageous for regenerative medicine and drug screening applications.

EXPERIMENTAL PROCEDURES

Experimental procedures are provided in the Supplemental Information.

Cell Culture, DE Differentiation, and Tissue Co-culture

Donors' informed consents were obtained for all experiments using human tissue, and all procedures involving the use of human cells and mice were approved by the ethics commission of Yokohama City University. In the absence of any mention, all cells were maintained at 37°C in a humidified atmosphere containing 5% CO_2 and 95% ambient air. The hiPSC line, TkDA3-4 (kindly provided by Dr. Nakauchi at University of Tokyo), was maintained on Laminin 511 E8 fragment (iMatrix-511, kindly provided by Nippi)-coated dishes in StemFit (Ajinomoto). The EGFP knocked-in to adeno-associated virus integration site 1 (AAVS1:EGFP) was used for live imaging analysis *in vitro*. The differentiation protocol for the induction of hiPSC-DEs was based on a previous report with some modifications (Kajiwara et al., 2012). Briefly, hiPSCs were seeded on a Laminin 511 E8-fragment-coated (iMatrix-511, kindly provided by Nippi) dish with RPMI-1640 (Wako) with 1% B-27 Supplement, serum free (Gibco) containing 100 ng/mL activin A (kindly provided by Ajinomoto) and 50 ng/mL Wnt3a (R&D Systems). The medium was used for 6 days. At cell seeding (D0), 10 μM ROCK Inhibitor Y-27632 (Wako) was added, and 1 mM sodium butyrate (Sigma) was added from D1 to D3. HUVECs and MSCs were cultured in endothelial cell growth medium (EGM; Lonza) or MSC growth medium (MSCGM; Lonza). For live imaging experiments, HUVECs were fluorescently labeled with KO1-encoding retroviruses constructed from

(E) ELISA of protein secretion in hiPSC-LBs cultured with various A83-01 concentrations for 15 days in Excess-hypoxia group (mean ± SD; n = 9–17 independent experiments; $^{*}p < 0.05$ and $^{**}p < 0.01$ versus 0 μM; $^{\S\S}p < 0.01$ versus 0.05 μM, $^{\#}p < 0.05$ versus 0.5 μM; $^{\#\#}p < 0.05$ versus 0.5 μM).
(F) Putative mechanism of O_2-dependent differentiation in human LBs through TGFB signaling.

a retrovirus vector pGCDNsam possessing a long terminal repeat derived from murine stem cell virus (MSCV). In brief, 293-gp and 293-gpg packaging cells were transfected with the retrovirus vector pGCDNsam-IRES KOFP (kindly provided by Dr Masafumi Onodera) and the culture supernatants were used for infection (Camp et al., 2017). Viable human adult hepatocytes (BioreclamationIVT; lots IVT-M00995) were cultured in the modified Lanford medium (Nissui) and albumin secretion were measured on the first 24 hr of culture. The mouse livers collected from E13.5 fetal mice were cut into block and culture for 72 hr onto 6.5-mm-diameter Transwell polycarbonate filter membranes (0.4 μm pore size; Corning) in a 24-wells plate holding 250 μL/well of medium under 10% O_2. The medium consisted of DMEM (high glucose) with L-glutamine and phenol red (Wako) containing 10% fetal bovine serum (biowest), 5% Matrigel (Corning), and 100 units/mL penicillin-streptomycin (Gibco). A Leica TCS SP8 confocal microscope (Leica Microsystems) was used for imaging analysis of fetal mouse liver. z axis height was equalized for comparison of each group. ImageJ software (NIH) was used to quantify.

Generation of Human iPSC-LBs

To generate hiPSC-LBs, we used micro-patterned plates made from O_2-permeable PDMS (Anada et al., 2012) or little permeable polystyrene. Total 9.0×10^5 cells of hiPSC-DE, HUVEC, and MSC were co-cultured on a well at the ratio of 10:7:2 in hepatocyte culture medium (HCM)/EGM medium. The medium is a 1:1 mixture of HCM (Lonza) without epidermal growth factor and EGM (Lonza) containing dexamethasone (0.1 mM, Sigma-Aldrich), oncostatin M (10 ng/mL, R&D Systems), hepatocyte growth factor (20 ng/mL, Kringle Pharma) and 2.5% fetal bovine serum, CELLect GOLD (MP Biomedicals). Only seeding time, 10 μM ROCK Inhibitor was added in the medium. To investigate the effect of O_2, hiPSC-LBs were cultured in 2%, 10%, 20%, and 40% O_2 during research periods. In TGFB signal inhibition research, 0 to 5 μM A83-01 (Wako) was added into culture medium for 15 days and 1 μM ROCK Inhibitor on the initial day. The diameters of collected hiPSC-LBs were measured by the IN-Cell Analyzer 2000 system (GE Healthcare). A Leica TCS SP8 confocal microscope (Leica Microsystems) was used for imaging analysis of hiPSC-LBs. z axis height and laser power were equalized for comparison of each groups. ImageJ software (NIH) was used to quantify average intensity of Kusabira-Orange fluorescence in hiPSC-LBs.

Microarray and scRNAseq

Gene expression data are downloaded and reanalyzed from the publicly available GEO, NCBI under accession numbers GEO: GSE46631, GSE81252, and GSE96981.

SUPPLEMENTAL INFORMATION

Supplemental Information includes Supplemental Experimental Procedures and four figures and can be found with this article online at https://doi.org/10.1016/j.stemcr.2018.06.015.

AUTHOR CONTRIBUTIONS

H.A. conceived the study, designed and performed the experiments, collected and analyzed the data, and wrote the manuscript. T.A., T.K., T.S., M.K., E.Y., S.K., A.F., Y.U., K.S., J.G.C., and B.T. performed and analyzed the experiments. T.T. conceived the study, designed the experiments, wrote and reviewed the manuscript, optimized the experiments, and supervised the study. O.S. and H.T. supervised the study.

ACKNOWLEDGMENTS

The authors would like to thank J. Sato for providing useful information regarding this project. The authors would also like to thank the members of our laboratory for their comments on the manuscript. This work was mainly supported through a grant from the Japan Science and Technology Agency: new area of Hyper Bio Assembler for 3D Cellular Systems to T.T. and O.S. (no. 24106510). This work was also supported by the AMED through its research grant “Center for Clinical Application Research on Specific Disease/Organ,” Research Center Network for Realization of Regenerative Medicine to H.T. This work was also supported by a Grant-in-Aid from the Ministry of Education, Culture, Sports, Science and Technology of Japan to T.A. (no. 17H02095). This work was also partly supported by PRESTO, Japan Science and Technology Agency (JST) to T.T. and Grants-in-Aid from the Ministry of Education, Culture, Sports, Science and Technology of Japan to T.T. (nos. 24106510 and 24689052). T.T. is a New York Stem Cell Foundation – Robertson Investigator.

Received: December 31, 2017
Revised: June 19, 2018
Accepted: June 20, 2018
Published: July 19, 2018

REFERENCES

Anada, T., Fukuda, J., Sai, Y., and Suzuki, O. (2012). An oxygen-permeable spheroid culture system for the prevention of central hypoxia and necrosis of spheroids. Biomaterials *33*, 8430–8441.

Anada, T., Sato, T., Kamoya, T., Shiwaku, Y., Tsuchiya, K., Takano-Yamamoto, T., Sasaki, K., and Suzuki, O. (2016). Evaluation of bioactivity of octacalcium phosphate using osteoblastic cell aggregates on a spheroid culture device. Regen Ther *3*, 58–62.

Antoniou, A., Raynaud, P., Cordi, S., Zong, Y., Tronche, F., Stanger, B.Z., Jacquemin, P., Pierreux, C.E., Clotman, F., and Lemaigre, F.P. (2009). Intrahepatic bile ducts develop according to a new mode of tubulogenesis regulated by the transcription factor SOX9. Gastroenterology *136*, 2325–2333.

Asai, A., Aihara, E., Watson, C., Mourya, R., Mizuochi, T., Shivakumar, P., Phelan, K., Mayhew, C., Helmrath, M., Takebe, T., et al. (2017). Paracrine signals regulate human liver organoid maturation from induced pluripotent stem cells. Development *144*, 1056–1064.

Augustin, H.G., Koh, G.Y., Thurston, G., and Alitalo, K. (2009). Control of vascular morphogenesis and homeostasis through the angiopoietin-Tie system. Nat. Rev. Mol. Cell Biol. *10*, 165–177.

Bascom, C.C., Wolfshohl, J.R., Coffey, R.J., Madisen, L., Webb, N.R., Purchio, A.R., Derynck, R., and Moses, H.L. (1989). Complex regulation of transforming growth factor beta 1, beta 2, and beta 3 mRNA expression in mouse fibroblasts and keratinocytes by

transforming growth factors beta 1 and beta 2. Mol. Cell. Biol. *9*, 5508–5515.

Bernhardt, W.M., Schmitt, R., Rosenberger, C., Münchenhagen, P.M., Gröne, H.J., Frei, U., Warnecke, C., Bachmann, S., Wiesener, M.S., Willam, C., et al. (2006). Expression of hypoxia-inducible transcription factors in developing human and rat kidneys. Kidney Int. *69*, 114–122.

Black, D., Lyman, S., Qian, T., Lemasters, J.J., Rippe, R.A., Nitta, T., Kim, J.S., and Behrns, K.E. (2007). Transforming growth factor beta mediates hepatocyte apoptosis through Smad3 generation of reactive O2 species. Biochimie *89*, 1464–1473.

Bohm, F., Kohler, U.A., Speicher, T., and Werner, S. (2010). Regulation of liver regeneration by growth factors and cytokines. EMBO Mol. Med. *2*, 294–305.

Byrne, M.B., Leslie, M.T., Gaskins, H.R., and Kenis, P.J. (2014). Methods to study the tumor microenvironment under controlled oxygen conditions. Trends Biotechnol. *32*, 556–563.

Camp, J.G., Sekine, K., Gerber, T., Loeffler-Wirth, H., Binder, H., Gac, M., Kanton, S., Kageyama, J., Damm, G., Seehofer, D., et al. (2017). Multilineage communication regulates human liver bud development from pluripotency. Nature *546*, 533–538.

Cazes, A., Galaup, A., Chomel, C., Bignon, M., Brechot, N., Le Jan, S., Weber, H., Corvol, P., Muller, L., Germain, S., et al. (2006). Extracellular matrix-bound angiopoietin-like 4 inhibits endothelial cell adhesion, migration, and sprouting and alters actin cytoskeleton. Circ. Res. *99*, 1207–1215.

Clotman, F., Jacquemin, P., Plumb-Rudewiez, N., Pierreux, C.E., Van der Smissen, P., Dietz, H.C., Courtoy, P.J., Rousseau, G.G., and Lemaigre, F.P. (2005). Control of liver cell fate decision by a gradient of TGF beta signaling modulated by Onecut transcription factors. Genes Dev. *19*, 1849–1854.

Dunwoodie, S.L. (2009). The role of hypoxia in development of the mammalian embryo. Dev. Cell *17*, 755–773.

Dye, B.R., Dedhia, P.H., Miller, A.J., Nagy, M.S., White, E.S., Shea, L.D., and Spence, J.R. (2016). A bioengineered niche promotes in vivo engraftment and maturation of pluripotent stem cell derived human lung organoid. Elife *5*. https://doi.org/10.7554/eLife.19732.

Ferrari, G., Terushkin, V., Wolff, M.J., Zhang, X., Valacca, C., Poggio, P., Pintucci, G., and Mignatti, P. (2012). TGF-β1 induces endothelial cell apoptosis by shifting VEGF activation of p38(MAPK) from the prosurvival p38β to proapoptotic p38α. Mol. Cancer Res. *10*, 605–614.

Gordillo, M., Evans, T., and Gouon-Evans, V. (2015). Orchestrating liver development. Development *142*, 2094–2108.

Hung, S.P., Yang, M.H., Tseng, K.F., and Lee, O.K. (2013). Hypoxia-induced secretion of TGF-β1 in mesenchymal stem cell promotes breast cancer cell progression. Cell Transplant *22*, 1869–1882.

Kajiwara, M., Aoi, T., Okita, K., Takahashi, R., Inoue, H., Takayama, N., Endo, H., Eto, K., Toguchida, J., Uemoto, S., and Yamanaka, S. (2012). Donor-dependent variations in hepatic differentiation from human-induced pluripotent stem cells. Proc. Natl. Acad. Sci. USA *109*, 12538–12543.

Kamoya, T., Anada, T., Shiwaku, Y., Takano-Yamamoto, T., and Suzuki, O. (2016). An oxygen-permeable spheroid culture chip (Oxy chip) promotes osteoblastic differentiation of mesenchymal stem cells. Sens. Actuators B Chem. *232*, 75–83.

Kretzschmar, K., and Clevers, H. (2016). Organoids: modeling development and the stem cell niche in a dish. Dev. Cell *38*, 590–600.

Lancaster, M.A., and Knoblich, J.A. (2014). Organogenesis in a dish: modeling development and disease using organoid technologies. Science *345*, 1247125.

Lin, T.Y., Chou, C.F., Chung, H.Y., Chiang, C.Y., Li, C.H., Wu, J.L., Lin, H.J., Pai, T.W., Hu, C.H., and Tzou, W.S. (2014). Hypoxia-inducible factor 2 alpha is essential for hepatic outgrowth and functions via the regulation of leg1 transcription in the zebrafish embryo. PLoS One *9*, e101980.

Nguyen, L.N., Furuya, M.H., Wolfraim, L.A., Nguyen, A.P., Holdren, M.S., Campbell, J.S., Knight, B., Yeoh, G.C., Fausto, N., and Parks, W.T. (2007). Transforming growth factor-beta differentially regulates oval cell and hepatocyte proliferation. Hepatology *45*, 31–41.

Sanchez, A., Alvarez, A.M., Pagan, R., Roncero, C., Vilaro, S., Benito, M., and Fabregat, I. (2000). Fibronectin regulates morphology, cell organization and gene expression of rat fetal hepatocytes in primary culture. J. Hepatol. *32*, 242–250.

Spagnoli, F.M., Cicchini, C., Tripodi, M., and Weiss, M.C. (2000). Inhibition of MMH (Met murine hepatocyte) cell differentiation by TGF (beta) is abrogated by pre-treatment with the heritable differentiation effector FGF1. J. Cell Sci. *113*, 3639–3647.

Sugiyama, D., Kulkeaw, K., and Mizuochi, C. (2013). TGF-beta-1 up-regulates extra-cellular matrix production in mouse hepatoblasts. Mech. Dev. *130*, 195–206.

Swartley, O.M., Foley, J.F., Livingston, D.P., III, Cullen, J.M., and Elmore, S.A. (2016). Histology atlas of the developing mouse hepatobiliary hemolymphatic vascular system with emphasis on embryonic days 11.5-18.5 and early postnatal development. Toxicol. Pathol. *44*, 705–725.

Takebe, T., Sekine, K., Enomura, M., Koike, H., Kimura, M., Ogaeri, T., Zhang, R.R., Ueno, Y., Zheng, Y.W., Koike, N., et al. (2013). Vascularized and functional human liver from an iPSC-derived organ bud transplant. Nature *499*, 481–484.

Takebe, T., Sekine, K., Kimura, M., Yoshizawa, E., Ayano, S., Koido, M., Funayama, S., Nakanishi, N., Hisai, T., Kobayashi, T., et al. (2017). Massive and reproducible production of liver buds entirely from human pluripotent stem cells. Cell Rep. *21*, 2661–2670.

Takehara, K., LeRoy, E.C., and Grotendorst, G.R. (1987). TGF-beta inhibition of endothelial cell proliferation: alteration of EGF binding and EGF-induced growth-regulatory (competence) gene expression. Cell *49*, 415–422.

Watson, C.L., Mahe, M.M., Munera, J., Howell, J.C., Sundaram, N., Poling, H.M., Schweitzer, J.I., Vallance, J.E., Mayhew, C.N., Sun, Y., et al. (2014). An in vivo model of human small intestine using pluripotent stem cells. Nat. Med. *20*, 1310–1314.

Xinaris, C., Benedetti, V., Rizzo, P., Abbate, M., Corna, D., Azzollini, N., Conti, S., Unbekandt, M., Davies, J.A., Morigi, M., et al. (2012). In vivo maturation of functional renal organoids formed from embryonic cell suspensions. J. Am. Soc. Nephrol. *23*, 1857–1868.

Zorn, A.M. (2008). Liver development. In StemBook, The Stem Cell Research Community., ed. (Harvard Stem Cell Institute), Available at. stembook.org/node/512.

Stem Cell Reports, Volume *11*

Supplemental Information

Optimal Hypoxia Regulates Human iPSC-Derived Liver Bud Differentiation through Intercellular TGFB Signaling

Hiroaki Ayabe, Takahisa Anada, Takuo Kamoya, Tomoya Sato, Masaki Kimura, Emi Yoshizawa, Shunyuu Kikuchi, Yasuharu Ueno, Keisuke Sekine, J. Gray Camp, Barbara Treutlein, Autumn Ferguson, Osamu Suzuki, Takanori Takebe, and Hideki Taniguchi

SUPPLEMENTAL INVENTORY

Optimal Hypoxia Regulates Human iPSC-Derived Liver Bud Differentiation Through Intercellular TGFB Signaling

Hiroaki Ayabe[1], Takahisa Anada[2], Takuo Kamoya[2], Tomoya Sato[2], Masaki Kimura[4], Emi Yoshizawa[1], Shunyuu Kikuchi[1], Yasuharu Ueno[1], Keisuke Sekine[1], J. Gray Camp[3], Barbara Treutlein[3], Autumn Ferguson[4], Osamu Suzuki[2], Takanori Takebe[1,4,5,6], Hideki Taniguchi[1]

Inventory of supplemental Information

Figure S1: This figure is related to Figure 1, 2 3 and 4.

Figure S2: This figure is related to Figure 1 and 2.

Figure S3: This figure is related to Figure 2 and 3.

Figure S4: This figure is related to Figure 3 and 4.

Supplemental Information

Supplemental Figure and Legends

A

O_2 condition	Air O_2 (%)	O_2 level (%)	Plate O_2 permeability
Extremely-hypoxia	10	8	Low
Excess-hypoxia	20	10	Low
Mild-hypoxia	10	13	High
Ambient	20	19	High
Hyperoxia	40	NA	Low
Strong-hypoxia	2	NA	High

B

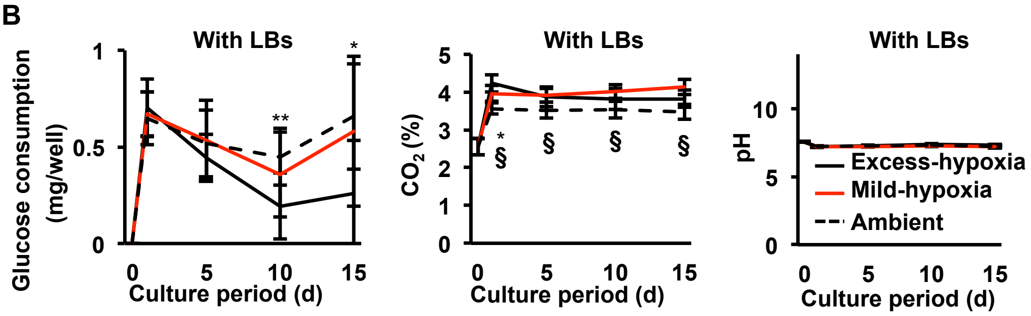

C

Test Group	n	Number (Liver buds/well)	Diameter (μm)	Median (μm)
Excess-hypoxia	11	353.5 ± 51.1	116.8 ± 8.9	122
Mild-hypoxia	9	489.3 ± 57.1 §§	139.3 ± 10.7 §§	143
Ambient	11	365.2 ± 96.8	110.4 ± 12.2	112

D

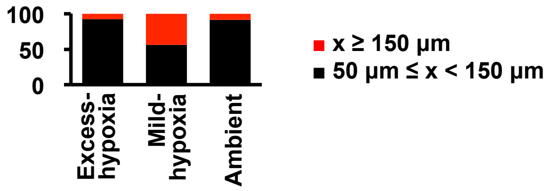

E

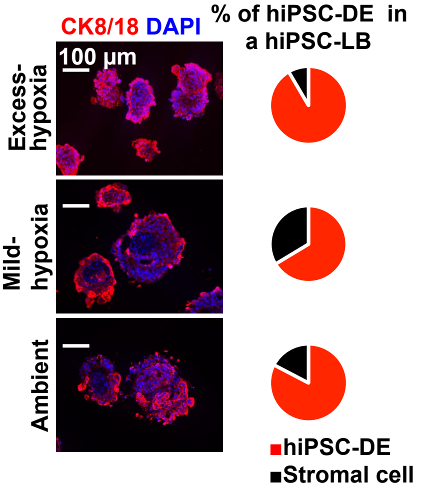

Figure S1. Test conditions of this study, environmental factor changes in hiPSC-LB culture on test set 1 and effect of O_2 in hiPSC-LB number/size. Related to Figure 1, 2, 3 and 4;

(A) Oxygen supply condition of hiPSC-LB cultured in this study. (B) Glucose consumption, CO_2 tension and pH change in culture medium with hiPSC-LB for 15 days (mean±SD; n=6~16 independent experiments; *: P<0.05 vs. Excess-hypoxia; §: P<0.05 vs. Mild-hypoxia). (C) Mean of hiPSC-LB number and diameter after 15 days culture (mean±SD; n=9~11 independent experiments; §§: P<0.01 vs. Mild-hypoxia). (D) Rate of over 150 μm hiPSC-LB in diameter after 15 days culture (From 3887 to 4404 hiPSC-LBs over 50 μm were tested). (E) Percentage of hiPSC-DE in a hiPSC-LB analyzed by immunohistochemistry on day15 (mean±SD; n=19~21; 4 independent experiments; CK8/18 (red) and Nuclei (DAPI; blue).

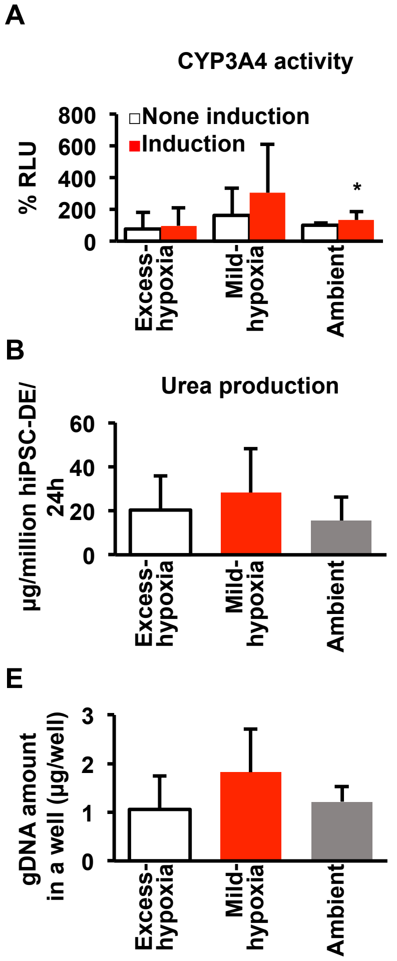

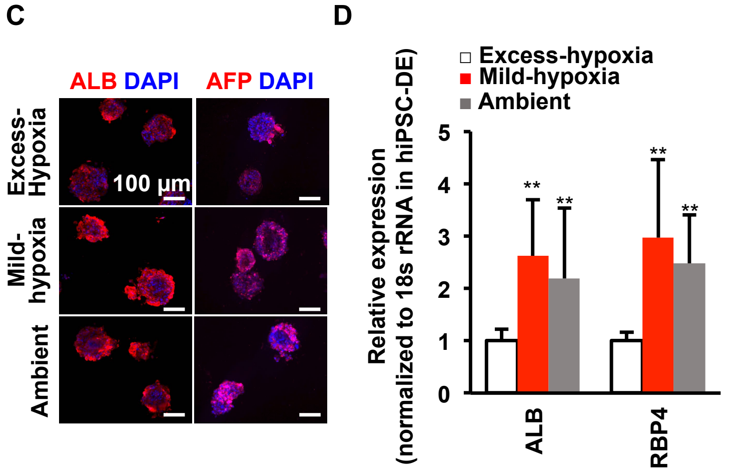

F

		Cells (x10E5)		
		hiPSC-DE	HUVEC, MSC	hiPSC-LB
Seeding Time	Day0	4.70	4.30	9.00
Excess-hypoxia	Day15	1.48±0.97	0.14±0.09	1.61±1.06
Mild-hypoxia	Day15	1.84±0.89	0.93±0.45	2.77±1.35
Ambient	Day15	1.53±0.39	0.32±0.08	1.85±0.48

Figure S2. Effect of O_2 in hiPSC-LB differentiation. Related to Figure 1 and 2;
(A) CYP3A4 activity in hiPSC-LB on day 20. Rifampicin was treated for 3 days. (mean±SD; n=11~13 independent experiments; *: P<0.05 vs. None induction). (B) Urea production in hiPSC-LB on day 20. NH_3Cl_4 was treated for 0 or 6 hours (mean±SD; n=11 independent experiments; **: P<0.01 vs. Excess-hypoxia). The values of urea production in a well were normalized by hiPSC-DE number in a well on day 15 (mean±SD; n=11 independent experiments). (C) IHC for ALB (red), AFP (red) and Nuclei (DAPI; blue) in hiPSC-LB on day15. Scale bar, 100 µm. (D) Relative gene expression in hiPSC-LB on day15. The gene expression levels were normalized to 18s rRNA expressed in hiPSC-DE. The ratio of each cell line was based on figure 2E (mean±SD; n=10 independent experiments; **: P<0.05 vs. Excess-hypoxia). (E) gDNA amount in a well on day15. mean±SD; n=10~11 independent experiments. (F) Cell number in a well on day 15 were calculated from hiPSC-DE and stroma (HUVEC and MSC) ratio in a hiPSC-LB Immunohistochemistry (IHC) and from gDNA amount in a well (6.57pg/cell; Serth et al., 2000).

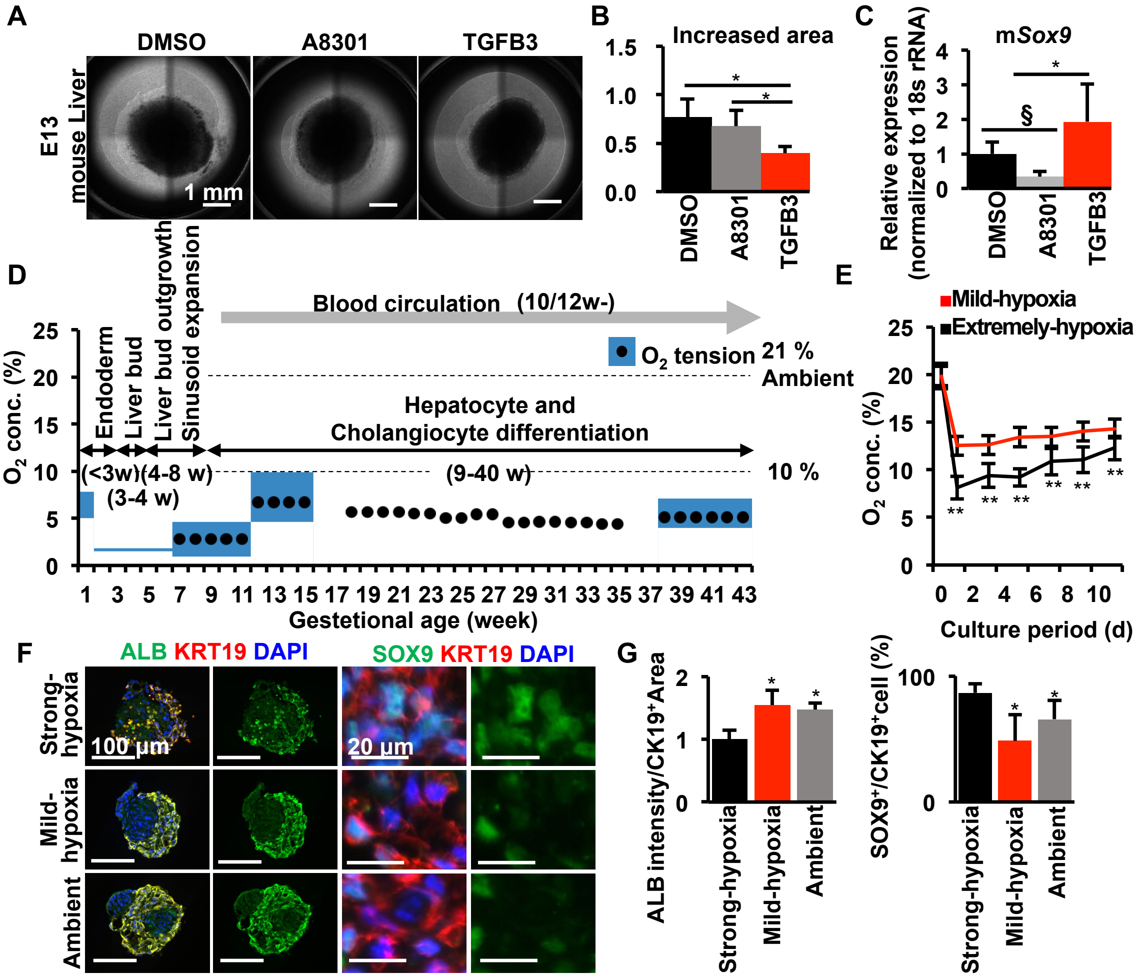

Figure S3. Effect of TGFB signal in fetal mouse liver, O_2 concentration change in human embryo developmental stage and O_2 dependent expression change in hepatocyte and cholangiocyte marker. Related to Figure 2 and 3; (A) Phase contrast imaging of E13.5 mouse liver tissue cultured with DMSO, 0.5 µM A83-01 or 10ng/mL TGFB3 for 72 hours (Scale bar, 1 mm). (B) Relative increased area of E13.5 mouse liver tissue cultured after 24 hours (mean±SD; n=6 independent experiments; *: P<0.05 vs. TGFB3). (C) Gene expression of E13.5 mouse liver tissue cultured after 72 hours (mean±SD; n=6 independent experiments; *: P<0.05 vs. TGFB3; §: P<0.05 vs. A83-01). (D) Change of oxygen exposure on human fetal development. Minimum and maximum oxygen concentrations are indicated blue belt. Mean oxygen concentrations are indicated black circle. Blood circulation is indicated gray line. Liver development events in each stage are indicated in the figure. This figure is created from various references (Tuuli et al., 2011; Morin 2017; Gregg et al., 1993; Gordillo et al., 2015). (E) Changes in O_2 concentration in culture medium with hiPSC-LBs (mean±SD; n=7~9 independent experiments; **P<0.01 vs. Mild-hypoxia). (F) Immunofluoresence staining for KRT19 (red), ALB (green) or SOX9 (green) in hiPSC-LB cultured for 5 days. Nuclei were stained using DAPI (blue). Scale bar, 100 µm (left side) and 20 µm (right side). (G) Analysis of hiPSC-LB in Figure S3F (mean±SD; n=6~8; 3 independent experiments; *: P<0.05 vs. Strong-hypoxia).

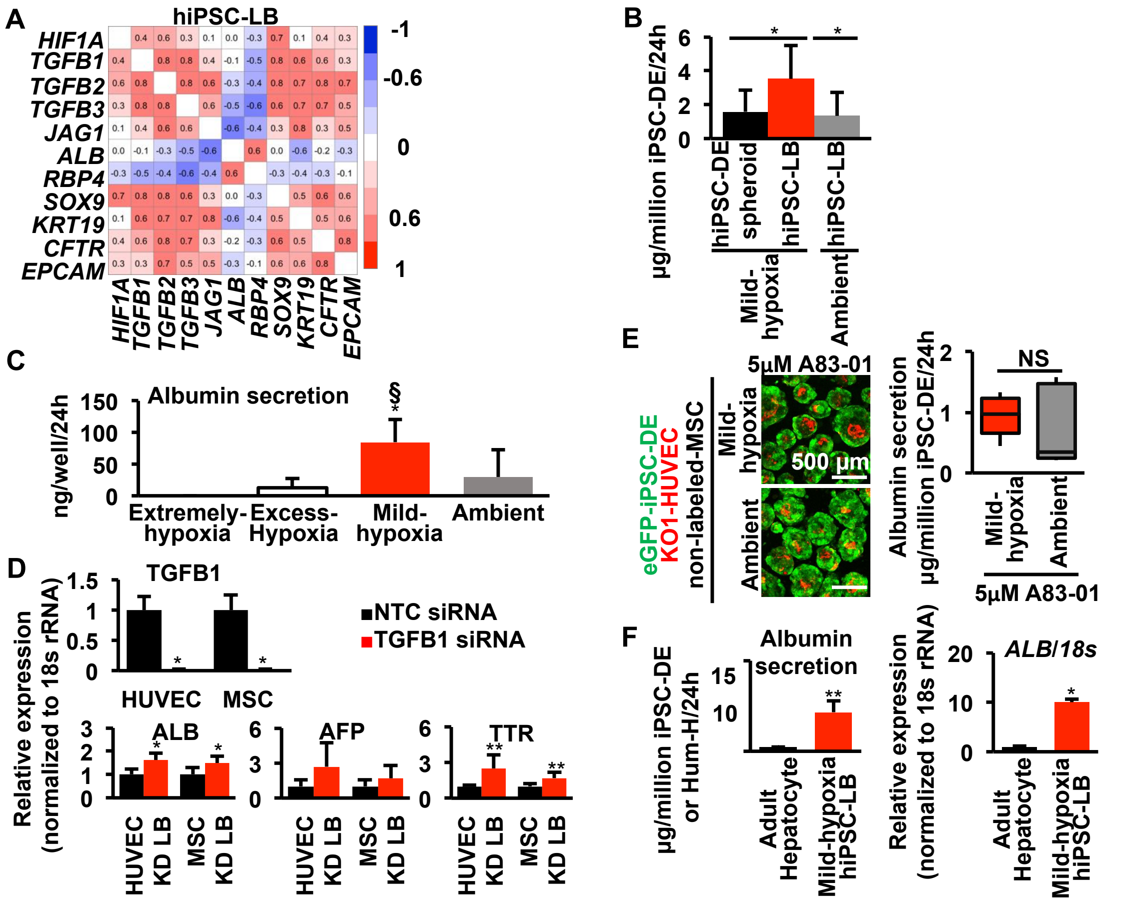

Figure S4. Correlation between O_2 and TGFB signal dependent expression change in hepatocyte and cholangiocyte markers, effect of stromal cells and O_2 in hiPSC-LB, and albumin level of adult hepatocyte. Related to Figure 3 and 4;
(A) Correlation analysis of hypoxia versus hepatocyte and cholangiocyte markers expressions in hiPSC-LB cultured for 10 days (n=36 independent experiments). (B) ELISA based albumin level of hiPSC-LB and hiPSC-DE spheroid cultured in Mild-hypoxia or Ambient for 15 days. The culture medium and growth factors are same as Figure 1E (mean±SD; n=7~22 independent experiments; *: P<0.05 vs. hiPSC-LB cultured in Mild-hypoxia). (C) Simultaneous comparison of albumin secretion of hiPSC-LBs cultured on day 10 in 4 conditions (mean±SD; n=8 or 9 independent experiments; * P<0.05 vs. Extremely-hypoxia; § P<0.05 vs. Excess-hypoxia). (D) Relative gene expression in TGFB1 knockdown (KD) study with siRNA treated-HUVEC/MSC and hiPSC-LB generated from TGFB1 KD HUVEC/MSC cultured on Excess-hypoxia (Top; mean±SD; n=6 independent experiments; *: P<0.05 vs. NTC siRNA, Bottom; mean±SD; n=8 independent experiments; *: P<0.05 and **: P<0.01 vs. hiPSC-LB generated from NTC siRNA treated HUVEC/MSC; NTC: Non-targeting control). (E) Inhibition of appropriate TGFB signal compensates O_2-dependent hepatic differentiation promotion in Mild-hypoxia (Left; Confocal images of hiPSC-LBs cultured in 5μM A83-01 for 15 days, green: eGFP-iPSC-DE cells (AAVS1::eGFP); red: KO1-HUVECs (MSCV-KO1); no label: MSCs; scale bar, 500 μm, Right; Boxplots of hiPSC-DE cell number normalized albumin level in hiPSC-LB on day15, The error bars represent the maximum and minimum values; n=12 independent experiments). The numbers of hiPSC-DE were calculated using the same rule as Figure 1E. (F) ELISA and gene expression based albumin level of adult hepatocyte and hiPSC-LB generated in Mild-hypoxia (Left; mean±SD; n=5or18 independent experiments; **: P<0.01 vs. hiPSC-LB generated in Mild-hypoxia; Right; n=5 or 6 independent experiments; *: P<0.05 vs. generated in Mild-hypoxia).

Supplemental Experimental Procedures

Animal

For mouse liver bud histology analysis, a female C57BL/6J mouse (SLC) on the tenth day of pregnancy was anesthetize with CO_2 and fetal mice before circulation (Swartley et al., 2016) were harvested rapidly for fixation. In the mouse liver tissue culture, female mice on the 13th day of pregnancy were anesthetized with CO_2 and fetal mice were harvested for liver dissection.

Measurement of pO_2, pCO_2, pH and glucose in hiPSC-LB culture medium

Partial pressure of oxygen (pO_2) in culture medium was measured by the Fibox3 single-channel fiber-optic oxygen meter (Presens) after 24 hours of last medium change. Partial pressure of carbon dioxide (pCO_2), pH and glucose consumption in culture medium were measured by Bioprofile 400 (kindly provided by Nova biomedical) after 24 hours of last medium change. We are not able to measure O_2 consumption by the cells due to the PDMS's higher O_2 diffusion coefficient. Therefore, we decided to measure dissolved O_2 in the medium instead of a direct measurement of O_2 consumption by the cells (Ramamoorthy et al., 2003, Kojima et al., 2015, Mattei et al., 2017 and Saito et al., 2006).

Immunostaining

hiPSC-LBs or mouse embryos were fixed in 4% paraformaldehyde (Wako) in phosphate buffered saline (PBS) for 15 minutes or overnight at 4 °C and infused in 30% sucrose (Wako) in PBS until sample sinks. Then the solution was replaced by 7.5% gelatin (Sigma) with 10% sucrose in PBS at 37°C for 15 minutes. The samples were embedded in new 7.5% gelatin with 10% sucrose in PBS and frozen with liquid N_2 after polymerization. Cryosections were placed on MAS-coated slides (Matsunami) for standard histological staining with hematoxylin/eosin (HE) or for immunostaining. For immunostaining, the samples were blocked by Protein Block Serum-Free (Dako), and were incubated with primary antibody at 4°C for overnight. Appropriate secondary antibodies conjugated to Alexa Fluor (Life Technologies) were incubated with the samples for 40 minutes at room temperature followed by DAPI (Sigma) nuclear staining. 0.05 % Tween20 (MP Biomedicals) in PBS was used for the sample washing and antibody dilution. The images were acquired using an Axio imager M1 microscope (Carl Zeiss). Image J software (NIH) was used to quantify the positive staining area.

Antibodies

Antibody	clone	Vendor	Catalog No.
Desmin		Lab Vision	RB-9014-P1
CD31	JC70A	Dako	M0823
Hif1a	H206	Santa Cruz Biotechnology	sc-10790
Hif2a		Novus Biologicals	NB100-122
Dlk	24-11	Medical & Biological Laboratories	D187-4
ALB		Sigma-Aldrich	A3293
AFP	C-19	Santa Cruz Biotechnology	sc-8108
KRT8/18		PROGEN	GP11
KRT19	RCK108	Dako	M0888
SOX9		EMD Millipore	AB5535

qRT-PCR and Microarray analysis

Total RNA was prepared using a PureLink RNA Mini Kit (Invitrogen). Then, qRT-PCR analyses and Microarray were conducted as described previously(Takebe et al., 2013). For qRT-PCR, total RNAs were reverse-transcribed with High-Capacity cDNA Reverse Transcription Kit (Applied Biosystems). 25ng of complementary DNAs (cDNAs) were used as qRT-PCR templates and mixed with gene specific primers and Taqman probe (Universal ProbeLibrary; Roche). Eukaryotic 18S rRNA Endogenous Control (Applied Biosystems) was used as reference and cycle reaction was performed using light cycler 480 systems (Roche). Gene expression levels were analyzed by ΔΔCp method. For microarray analysis, RNAs extracted from CD45-negative and Ter119-negative murine liver cells were hybridized on Whole Mouse Genome Agilent 4 X 44K v2 Oligonucleotide Microarray (Agilent Technologies) according to the manufacturer's instructions. Gene expression levels were analyzed using GeneSpring11.5.1.

qRT-PCR primers

Primer		sequence 5'>3'	Roche probe ID
ALB	Left	AATGTTGCCAAGCTGCTGA	27
	Right	CTTCCCTTCATCCCGAAGTT	
RBP4	Left	CCAGAAGCGCAGAAGATTG	17
	Right	TTTCTTTCTGATCTGCCATCG	
TGFB1	Left	CGACTACTACGCCAAGGAGGT	31
	Right	TGCTTGAACTTGTCATAGATTTCG	
TGFB2	Left	CAAAGGGTACAATGCCAACTT	67
	Right	GCAGATGCTTCTGGATTTATGG	
TGFB3	Left	AAGAAGCGGGCTTTGGAC	38
	Right	CGCACACAGCAGTTCTCC	
JAG1	Left	GGGCAACACCTTCAACCTC	28
	Right	GCCTCCACAAGCAACGTATAG	
INHBA	Left	CTCGGAGATCATCACGTTTG	72
	Right	CCTTGGAAATCTCGAAGTGC	
ANG1	Left	GACAGATGTTGAGACCCAGGTA	67
	Right	TCTCTAGCTTGTAGGTGGATAATGAA	
ANG2	Left	TGCAAATGTTCACAAATGCTAA	75
	Right	AAGTTGGAAGGACCACATGC	
ANGPTL4	Left	GTGGACCCTGAGGTCCTTC	18
	Right	CCACCTTGTGGAAGAGTTGC	
NODAL	Left	GGCGAGTGTCCTAATCCTGT	52
	Right	GCTGGTAACGTTTCAGCAGACT	

qRT-PCR primers

Primer		sequence 5'>3'	Roche probe ID
SOX9	Left	GTACCCGCACTTGCACAAC	61
	Right	TCTCGCTCTCGTTCAGAAGTC	
KRT19	Left	GCCACTACTACACGACCATCC	71
	Right	CAAACTTGGTTCGGAAGTCAT	
CFTR	Left	GAAGTAGTGATGGAGAATGTAACAGC	52
	Right	GCTTTCTCAAATAATTCCCCAAA	
HIF1A	Left	GAACCTGATGCTTTAACTTTGCT	28
	Right	TGCTGGTCATCAGTTTCTGTG	
EPCAM	Left	CCATGTGCTGGTGTGTGAA	3
	Right	TGTGTTTTAGTTCAATGATGATCCA	
mSox9	Left	GTACCCGCATCTGCACAAC	66
	Right	CTCCTCCACGAAGGGTCTCT	
VEGFA	Left	CGCAAGAAATCCCGGTATAA	1
	Right	AAATGCTTTCTCCGCTCTGA	

Albumin, AFP, Vitronectin, Ure, CYP3A4 and Genomic DNA measurement

To measure protein secretion level, HCM/EGM medium used for hiPSC-LB culture was collected after 24 hours from last medium change, and human Albumin Enzyme-Linked Immuno Sorbent Assay (ELISA) Quantitation Kit (Bethyl Laboratories), AFP (Human) ELISA Kit (Abnova) and Human Vitronectin ELISA Pair Set (Sino Biological) were used by according to the manufacturer's instructions. Absorbances of each sample were read by microplate reader, DTX880 (Beckman Coulter). On urea production assay, hiPSC-LBs were cultured in HCM/EGM medium for 16 days, and the medium was replaced to RPMI1640 including 80 ng/ml hepatocyte growth factor (HGF, Kringle Pharma), 2% B-27 Supplement, serum free, 1% MEM Non-Essential Amino Acids Solution and 100u/mL Penicillin-Streptomycin for next 4 days. Then the hiPSC-LBs were collected and cultured in the new medium with 2 mM NH_4Cl (Wako) on 96 wells plate. The medium was collected at 0 hrs and 6 hrs after hiPSC-LB re-seeding. Urea concentration in the medium was decided using QuantiChrom Urea Assay Kit (BioAssay Systems) used by according to the manufacturer's instructions. Absorbances of each sample were read by microplate reader, DTX880. To detect CYP3A4 activity, hiPSC-LBs were cultured in HCM/EGM medium for 15 days, and the medium was replaced to RPMI1640 including 80 ng/ml HGF, 2% B-27 Supplement, serum free, 1% MEM Non-Essential Amino Acids Solution and 100u/mL Penicillin-Streptomycin for next 2days.The medium was replaced to new medium with or without 25 μM Rifampicin (Wako) and cultured for 3 days. Then the hiPSC-LBs were collected and cultured in DMEM (high glucose) with L-glutamine and phenol red with 100u/mL Penicillin-Streptomycin, 0.1% CYP3A4-IPA (Promega), 1%

MEM Non-Essential Amino Acids Solution and 2% B-27 Supplement, serum free. The medium was collected after 6 hours and CYP3A4 activity was decided by CYP3A4 kit (Promega) used by according to the manufacturer's instructions. Luminescence of each sample were read by microplate reader, ARVO MX (Perkin Elmer). For genomic DNA quantification, collected hiPSC-LBs were incubated with 200mg/L Papain solution (Sigma-Aldrich) over night. Genomic DNAs in sample lysate were quantified by Quant-iT PicoGreen dsDNA Reagent (Invitogen) according to the manufacturer's instructions. Fluorescences of each sample were read by microplate reader, DTX880.

Sorting of hiPSC-LB

For performing fluorescence activated cell sorting (FACS), EGFP labeled iPSC-DE, KO1-labeled HUVEC and non-labeled MSC were co-cultured to generate hiPSC-LB. After 2 days, three wells of hiPSC-LBs (total 2.7E6 cells at the time of seeding) were pooled and wash with 1xPBS. The hiPSC-LBs were incubated with 2 mL of Accutase (Innovative Cell Technologies) about 1 hour at 37°C until cells were dissociated. Then the samples were centrifuged and re-suspended in 1mL of HCM/EGM. The dissociated cells were filtered with 35 µm nylon mesh (Falcon) and FACS-sorted by MoFlo Astrios (Beckman Coulter).

scRNA seq

The data were obtained from our previously published article (Camp et al., 2017) and can be downloaded from the NCBI Gene Expression Omnibus (GSE81252 and GSE96981). In brief, single cells dissociated from hiPSC-LB were captured on a microfluidic chip for mRNA-seq (Fluidigm) using a C1 system (Fluidigm), and SMARTer Ultra Low RNA kit for Illumina (Clontech) were used for cDNAs preparation on the chip. ERCC (External RNA Controls Consortium) RNA spike-in Mix (Invitrogen) was added to the lysis reaction and to process cellular mRNA at the same time. Sequencing libraries were constructed using a Nextera XT DNA Sample Preparation kit (Illumina) and each cell was sequenced using HiSeq 2500 (Illumina). Raw reads were mapped using TopHat and cDNAs were quantified as FPKM generated by Cufflinks. R script was used to perform PCA (FactoMineR package) and to construct violin plots.

TGFB1 Knock Down study

For TGFB1 knockdown study, 120 pmol of siRNA and 20 µL of Lipofectamine RNAiMAX Transfection Reagent (Thermo Fisher Scientific) were mixed in 2 mL of Opti-MEM I Reduced Serum Medium (Thermo Fisher Scientific) on 100 mm dishes and incubate for 20 minutes at room temperature. Then, 1.5E6 cells of HUVEC or 4.0E5 cells of MSC suspended in 8 mL of EGM (Lonza) or MSCGM (Lonza) without antibiotics were seeded before one day of co-culturing. These cells were co-cultured with hiPSC-DE for generating hiPSC-LB. After 4 days, these hiPSC-LBs were collected and quantified hepatic gene expression levels by qPCR analysis. The siRNAs used this study are siGENOME Non-Targeting siRNA Pool #2 (Dharmacon) or SMARTpool: siGENOME TGFB1 siRNA (Dharmacon).

Statistical Analysis

Mann-Whitney *U*-test was performed for 2 groups comparison. For multiple comparison, Mann-Whitney *U*-test with Bonferroni correction was performed after Kruskal Wallis

test. Pearson's correlation was performed for verifying 2 groups correlation. Two-tailed p value of < 0.05 was considered as significant. Data are expressed as the mean±SD (Additional details were described in figure legends)

Supplemental References

Gregg, A.R., and Weiner, C.P. (1993) "Normal" umbilical arterial and venous acid-base and blood gas values. Clin Obstet Gynecol. 36, 24-32

Iwama, A., Oguro, H., Negishi, M., Kato, Y., Morita, Y., Tsukui, H., Ema, H., Kamijo, T., Katoh-Fukui, Y., Koseki, H. and van Lohuizen, M., (2004) Enhanced self-renewal of hematopoietic stem cells mediated by the polycomb gene product Bmi-1. Immunity, 21, 843-851.

Kojima, M., Takehara, H., Akagi, T., Shiono, H., and Ichiki, T. (2015) Flexible sheet-type sensor for noninvasive measurement of cellular oxygen metabolism on a culture dish. PloS one, 10, e0143774.

Mattei, G., Magliaro, C., Giusti, S., Ramachandran, S. D., Heinz, S., Braspenning, J., & Ahluwalia, A. (2017). On the adhesion-cohesion balance and oxygen consumption characteristics of liver organoids. PloS one 12, e0173206.

Morin, S.J. (2017) Oxygen tension in embryo culture: does a shift to 2% O2 in extended culture represent the most physiologic system? J Assist Reprod Genet. 34, 309-314

Ramamoorthy, R., Dutta, P. K., and Akbar, S. A. (2003) Oxygen sensors: materials, methods, designs and applications. Journal of materials science 38, 4271-4282.

Saito, T., Wu, C. C., Shiku, H., Yasukawa, T., Yokoo, M., Ito-Sasaki, T., Abe H., Hoshi H. and Matsue T. (2006) Oxygen consumption of cell suspension in a poly (dimethylsiloxane)(PDMS) microchannel estimated by scanning electrochemical microscopy. Analyst 131, 1006-1011

Serth, J., Kuczyk, M.A., Paeslack, U., Lichtinghagen, R. and Jonas, U. (2000) Quantitation of DNA extracted after micropreparation of cells from frozen and formalin-fixed tissue sections. The American journal of pathology, 156, 1189-1196.

Tuuli, M.G., Longtine, M.S., and Nelson, D.M. (2011) Review: Oxygen and trophoblast biology-a source of controversy. Placenta 32, S109-S118
